# Modeling Lipid Vesicles’ Dynamic Response to Osmotic Pressure to Quantify Small-Molecule Transport

**DOI:** 10.1021/acs.langmuir.6c02777

**Published:** 2026-09-29

**Authors:** Sara Boi, Tiziana Podda, Alessandra Murroni, Stefan Milenkovic, Matteo Ceccarelli, Mariano Andrea Scorciapino

**Affiliations:** † 3111 University of Cagliari, Department of Chemical and Geological Sciences, Monserrato, Cagliari I-09042, Italy; ‡ 3111 University of Cagliari, Department of Life and Environmental Sciences - Section of Drug Sciences, Monserrato, Cagliari I-09042, Italy; § 3111 University of Cagliari, Department of Physics, Monserrato, Cagliari I-09042, Italy; ⊥ Université Paris-Saclay, Université Evry, CY Cergy Paris Université, CNRS, LAMBE, Evry-Courcouronnes, 91025, France

## Abstract

Lipid membrane permeability is fundamental for cellular homeostasis, signaling, and drug transport. Experimental methods exist to characterize it, from cell-based assays to more reproducible artificial, filter-supported systems. Although they closely mimic physiological environments, these assays pose challenges for quantitative analysis. Simpler models, such as supported lipid bilayers and liposomes, offer better control of experimental variables and membrane composition, making them suitable for detailed physicochemical investigations. The dynamic response of unilamellar lipid vesicles to osmotic pressure can be monitored via fluorescence spectroscopy, exploiting specific fluorophores, or label-free, by light scattering, which are among the most commonly used techniques. Proper analysis of the results allows the determination of permeant species’ permeability coefficients. A numerical algorithm was developed to simulate label-free liposome swelling/shrinking assays by explicitly considering the permeant’s water/membrane partition equilibrium and diffusion through the lipid membrane, besides the elastic properties of the latter. Optical density kinetics were collected for gradients of potassium chloride and a series of molecules with different permeability coefficients. The simulation code (adaptable also to fluorescence data) was iteratively coupled to a least-squares curve-fitting script to retrieve the permeability coefficient. The algorithm proved reliable, yielding results consistent with the literature. It represents both an analysis method and a useful screening tool, as shown by a series of numerical experiments. The study demonstrated a remarkable difference in sensitivity between swelling and shrinking directions. Although foreseen by theory, this system response asymmetry is not always included in other models, whereas it can make a great difference and must be considered when setting up experiments.

## Graphical abstract

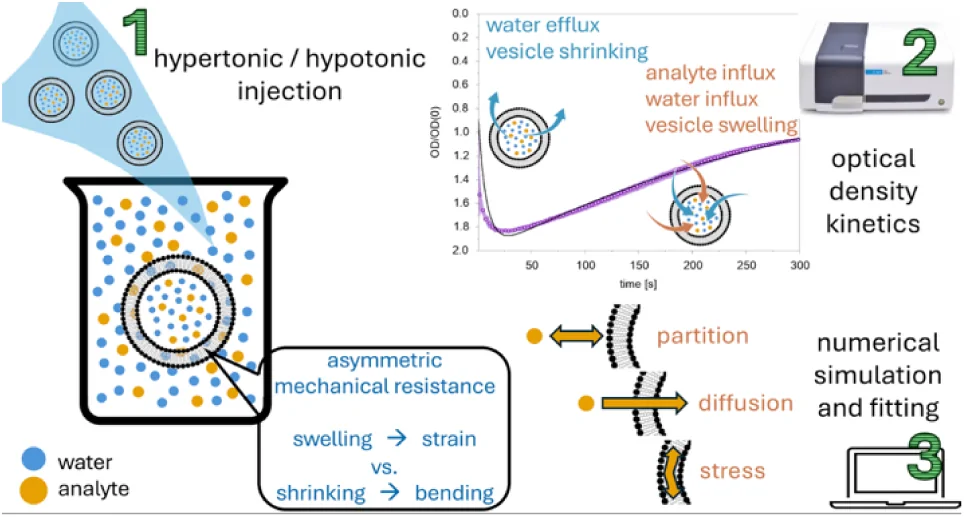

## Introduction

Biological membranes compartmentalize cells and organelles, regulating the flux of substances essential for homeostasis. Beyond physiological functions, membrane permeability dictates nutrient absorption and drug delivery, as therapeutic efficacy often depends on a molecule’s ability to partition into or penetrate biomembranes.^1–4^ Permeability is governed by membrane fluidity (modulated by lipid composition and temperature) and the physicochemical properties of the permeant, such as size, shape, and charge.^5,6^ While active transport requires metabolic energy, passive transport (e.g., simple diffusion or osmosis) is an exergonic process. Transport mechanisms are further classified by their electrogenic nature: net charge translocation through selective ion channels generates a membrane potential,^7,8^ whereas nonelectrogenic transport, such as glucose diffusion,^9^ does not change, nor is it affected by, the membrane potential. Transport stoichiometry is classified as uniport or cotransport (symport/antiport).^5^

In order to design molecular systems able to effectively cross a specific biological membrane, measuring and predicting permeability is essential. Cell-based methods, including tissue samples or whole-cell systems such as Caco-2 ^10,11^ or MDCK^12^ cell lines, closely resemble the physiological environment. Active transport systems are present, together with the potential for metabolic processing of the investigated substrates. Such inherent complexity, although valuable, makes a direct quantitative correlation between permeability and the permeant’s physicochemical characteristics highly challenging. In addition, large interlaboratory variability has been reported, which depends primarily on cell line origin and culture conditions.^12^

The PAMPA (Parallel Artificial Membrane Permeation Assay)^13–15^ or PVPA (Phospholipid Vesicle-based Permeation Assay)^12^ are highly reproducible and versatile alternatives in terms of lipid composition, making them particularly suitable for high-throughput screening campaigns. However, incorporating transport systems or using multicomponent lipid mixtures can be challenging, and isolating the specific contribution of the different physicochemical parameters remains difficult. Furthermore, these filter-supported systems are influenced by the support layer characteristics, which can complicate mechanistic interpretation.

Conversely, transport mechanisms have been extensively studied using simpler artificial models such as supported lipid bilayers^16^ and liposomes.^17^ These systems offer precise control over experimental variables, including lipid composition and protein incorporation, while providing high repeatability and reproducibility. Despite their simplified nature, liposomes allow for systematic, single-parameter studies. When combined with diverse analytical methods^18–27^ and theoretical frameworks,^20,24–26,28–38^ they are suitable for mechanistic physicochemical investigations and are capable of encapsulating both hydrophilic and lipophilic compounds across a wide range of sizes.^3,39,40^

In this work, we utilize the liposome swelling/shrinking assay (LSA),^19,20,24,26,34,41^ where vesicle volume changes are monitored spectroscopically to assess passive transport across the lipid bilayer. While many established protocols employ encapsulated fluorophores to achieve high sensitivity, such approaches introduce several complications, including potential fluorophore-membrane interactions, reporter leakage, or the necessity of purification steps to remove unencapsulated dye. Consequently, optical density (OD) measurements, which leverage vesicle size-dependent light scattering, emerge as an accessible, label-free alternative with streamlined sample preparation. Current data analysis for label-free LSA typically compares the initial slope of the kinetic curves.^20,41,42^ These practical methods often rely on linearized empirical relationships to estimate relative permeability coefficients (RPCs). While highly effective for molecular ranking, such approximations are optimized for initial-rate conditions rather than full system evolution. Ghosh and Singh introduced a pioneering mathematical model to shift toward full-curve analysis, proposing an exponential relationship to convert volume to absorbance.^20^ Light-scattering theories, such as the Rayleigh-Gans-Debye (RGD) approximation, provide a rigorous, nonlinear conversion of volume to OD.^26^

The present goal is to expand the quantitative analysis of label-free OD-LSA by introducing a robust numerical algorithm to extract absolute transport descriptors. This offers the possibility of producing coherent data sets that can be exploited to build predictive quantitative structure–property relationships (QSPR) and artificial intelligence models.^43^ We present a simple discrete-time numerical algorithm to simulate the vesicle response to osmotic pressure and passive uniport. Unlike existing transport models, our approach explicitly accounts for the water-membrane partition equilibrium and through-lipid diffusion, while incorporating the lipid bilayer’s elastic properties. By utilizing discrete time-step iterations rather than case-specific analytical equations, the algorithm remains highly flexible and directly extendable to complex scenarios involving charge gradients, acid–base equilibria, multiple permeants, and protein channels.^37^

The model was tested by using LSA performed on a series of molecules with different permeability coefficients, alongside the nonpermeating case of potassium chloride. By coupling the simulation code with a curve-fitting script, transport coefficients were successfully extracted. The experiments revealed a remarkable sensitivity asymmetry between the swelling and the shrinking regimes, a physical phenomenon captured by the numerical model. Finally, we demonstrated the code’s performance through numerical simulations, presenting it as a versatile analysis and screening tool for systematic transport studies.

## Experimental Section

The osmolarity of the solutions was determined with OsmoSpecial-1 (Astori Tecnica, Poncarale, IT), using the freezing point method. All the solutions were passed through Luer-lock PTFE syringe filters with 200 nm pores to remove possible dust before use. Lipid vesicles were prepared with 1-palmitoyl-2-oleoyl-glycero-3-phosphocholine (POPC; Avanti Polar Lipids, Alabaster, AL, USA). Lipids were weighed on an analytical balance and then solubilized in a 1:1 v/v CH_3_OH/CHCl_3_. The solvent was evaporated under a gentle nitrogen stream and then under vacuum overnight. The dry lipid film was hydrated with an appropriate aliquot of the desired solution (about 1 mL) to achieve a final lipid concentration of 14.7 mg/mL. The latter will be hereafter referred to as the “internal solution”. The lipid suspension was vortexed for 1 min and then passed 11 times through a polycarbonate filter with 400 nm pores using the Avanti Polar mini-extruder (Avanti Polar Lipids, Alabaster, AL, USA). Finally, the vesicle suspension was passed 21 times through a polycarbonate filter with 100 nm pores to obtain a homogeneous large unilamellar vesicle (LUV) suspension. The latter was characterized by dynamic light scattering (DLS) measurements, performed with the Zetasizer Nano (Malvern Panalytical, Malvern, UK). An aliquot of 60.0 μL of the vesicle stock solution was diluted into 1.0 mL of the same internal solution in a disposable cuvette measuring 1 × 1 cm. Three measurements were performed at 300 K, each comprising 12 runs of 10 s. The refractive index was set to 1.381 ^44^ and the dispersant viscosity to 0.860 mPa·s.^45^ For each LUV batch, the average vesicle diameter was found in the range 116–135 nm (with an average standard deviation, SD, among the three replicates of 3.2 nm), a size-distribution SD of 20–30 nm, and a polydispersity index below 0.1. Lipid concentration was determined through ^1^H NMR (PULCON),^46^ by drying an aliquot of the LUV sample under vacuum and then resuspending it in a known volume of CDCl_3_.

For the LSA, the optical density (OD) measurements were performed at 300 K with the Cary 60 UV–vis spectrophotometer (Agilent Technologies, Santa Clara, CA, USA), equipped with temperature and agitation control (NuovaCM, Orani, NU, Italy). An aliquot of 2.0 mL of the analyte solution, hereafter referred to as the “external solution”, was placed in a macro disposable cuvette with a 1 cm optical path length. The reading at a wavelength of 400 nm was set to zero, and OD kinetics were started. Then, 150.0 μL of the LUV stock solution was injected into the cuvette. Kinetics were recorded for 5 min, at 1 s intervals, with a spectral bandwidth of 1.5 nm. Each experiment was repeated 3 to 6 times. Results are shown (and analyzed) as the mean curve with the corresponding point-by-point SD. Starting concentrations are provided in the text and/or in the figure captions, with the starting gradient being 20–25 mOsm/L. For each sample, the “blank” experiment was always performed as a reference, i.e., by injecting the same aliquot of LUV into the internal solution itself, in order to acquire the OD kinetics corresponding to isotonic conditions. The pH of all samples, before and after measurements, was checked at 300 K by potentiometry, with the Hamilton (Reno, NV, USA) glass SpinTrode connected to an inoLab Level 1 pH meter (Weilheim, Germany), calibrated against three reference solutions at pH 4, 7, and 10, respectively.

## Numerical Model

The first step in simple uniport is the water-membrane partition equilibrium. The second step is diffusion through the lipid membrane. Eventually, the species will reach partition equilibrium on the other side of the membrane. The process is numerically divided into short, discrete time steps (1 μs each), and the molar amounts on both sides are iteratively updated up to the desired simulation time. Partition equilibria are assumed to be much faster than diffusion through the membrane, such that, as soon as the amount of each species is updated, equilibria are instantaneously reestablished.

When chemical equilibria in dilute solutions are expressed through their equilibrium constants, treating the solvent concentration as constant and, as such, incorporating it into the equilibrium constant is valid. However, this approximation cannot be applied to partition equilibria:^47^

1 $$S_{s}+L⇄S_{m}+\mathrm{W;},K_{p}=\frac{{[S]}_{m}\left[W\right]}{{[S]}_{s}\left[L\right]}$$

In eq 1, S_s_ and S_m_ are the molar concentrations of the solute in solution and in the membrane, respectively; L represents the lipids, and W represents the water. Due to practical difficulties in determining the thermodynamic partition equilibrium constant, K_p_, the partition coefficient p (lowercase is used in this work to distinguish it from permeability coefficients) can be conveniently used to compare the lipophilicity of different compounds.^47^ The latter is available through many databases and cheminformatics tools.^48–51^ We used ChemAxon’s Chemicalize (https://chemicalize.com; Budapest, Hungary, accessed June 2026) to evaluate p and the other geometric descriptors mentioned below. The relationship between the thermodynamic K_p_ and the empirical p is presented in the literature:^52,53^

2 $${p=K}_{p}\frac{\gamma_{W}}{\gamma_{L}}$$

where γ is the molar volume of the water or lipid molecules.

The molar concentration of the solute in the membrane-bound state is then given by

3 $${[S]}_{m}{=p\gamma }_{L}{[S]}_{s}\left[L\right]$$

where [S]_s_ is the molar concentration of S in solution at equilibrium, which can be obtained by solving:

4 $${[S]}_{s}\left({1+p\gamma }_{L}\left[L\right]\right)-{[S]}_{\mathrm{tot}}=0$$

with [S]_tot_ being the total S molar concentration.

To describe the diffusion of S through the lipid membrane, the homogeneous diffusion model,^54^ and thus, Fick’s law, is applied, calculating the flux density as

5 $$J_{S}=-\frac{D}{\Delta x}\Delta {[S]}_{m}\times 10^{3}$$

where D is the diffusion coefficient of S through the membrane, Δ­[S]_m_ is the concentration gradient across the membrane, and Δx is its hydrophobic thickness. Multiplication by 10^3^ is needed to change concentration from mol L^–1^ to mol m^–3^, giving flux density in mol m^–2^ s^–1^. In eq 5, it is worth noting that the partition coefficient is implicitly included through the calculation of the membrane-bound S and the corresponding gradient. Thus, the permeability coefficient of S is

6 $$P_{S}=\frac{D}{\Delta x}\left(\frac{{p\gamma }_{L}\left[L\right]}{{1+p\gamma }_{L}\left[L\right]}\right)$$

It is difficult to measure D in an anisotropic system, such as a lipid vesicle suspension. D is determined as a fitting parameter during the analysis of the experimental data, and then P_s_ is calculated with eq 6.

The flux density was iteratively multiplied by the vesicle’s total area, A_v_, and the time step, Δt, to determine the mole variation:

7 $$n_{S}\left(t\right){=n}_{S}\left(t-\Delta t\right)+J_{S}A_{v}\Delta t$$

Vesicles were modeled as spheres,^32,35,37,55–57^ with a diameter d given by the average value obtained from dynamic light scattering (DLS) measurements. To simplify the numerical treatment, the liposome population was modeled by using the mean vesicle diameter. This is justified by the very low polydispersity of our samples (<0.1) and their narrow size distribution. The potential impact of neglecting the vesicle size distribution on the simulation output was evaluated through a series of numerical experiments (Figure S1 and S2). To quantitatively estimate the error introduced by this simplification, a composite ensemble optical density curve was generated by calculating the weighted average of the individual optical responses across the full, binned experimental size distribution. This composite profile was then fitted using the single mean diameter value, showing an error of only −5.9% in the extracted diffusion coefficient (see the SI). In agreement with a previous report,^35^ we found that the mean value can be used to capture vesicle volume kinetics, unless the size distribution is remarkably asymmetric. The size distribution is worth including in a future version of the algorithm.

Total vesicle internal volume, V_in_, and the internal and external area, A_in_ and A_ex_, were estimated with the eqs 8–10, respectively:

8 $$V_{\mathrm{in}}=\frac{\left[L\right]V_{\mathrm{tot}}N_{A}{\left(\frac{d}{2}-\Delta x\right)}^{3}}{{3\sigma }_{L}\left[{\left(\frac{d}{2}\right)}^{2}+{\left(\frac{d}{2}-\Delta x\right)}^{2}\right]}\times 10^{3}$$

9 $$A_{\mathrm{in}}=\frac{\left[L\right]V_{\mathrm{tot}}N_{A}}{\sigma_{L}}\frac{{\left(\frac{d}{2}-\Delta x\right)}^{2}}{\left[{\left(\frac{d}{2}\right)}^{2}+{\left(\frac{d}{2}-\Delta x\right)}^{2}\right]}$$

10 $$A_{\mathrm{ex}}=\frac{\left[L\right]V_{\mathrm{tot}}N_{A}}{\sigma_{L}}\frac{{\left(\frac{d}{2}\right)}^{2}}{\left[{\left(\frac{d}{2}\right)}^{2}+{\left(\frac{d}{2}-\Delta x\right)}^{2}\right]}$$

where V_tot_ is the total sample volume, N_A_ is Avogadro number, and σ_L_ is the lipid surface density, which was previously measured for the POPC monolayer^56^ in agreement with other reports.^59,60^ The difference between the areas of the external and internal leaflets of the lipid bilayer was taken into account by using $\left(\frac{d}{2}-\Delta x\right)$ as the internal radius. The 10^3^ factor was needed to convert from m^3^ to L units. Either the external or the internal area is used in eq 7, depending on the sign of the flux density.

The total solute concentration on each side, at each time step, [S]_tot_, together with the total concentration of additional nonpermeating osmolytes, [O]_tot_, were used to iteratively calculate the osmolarity gradient, Δ_osm_. According to the van’t Hoff equation:

11 $${\Delta \Pi =\Delta }_{\mathrm{osm}}\times \mathrm{RT}\times 10^{3}$$

where the osmotic pressure difference, ΔΠ, is given by the product of the osmolarity gradient, the universal gas constant, and temperature. Multiplication by 10^3^ was needed to change the concentration from mol L^–1^ to mol m^–3^. This formulation (and all the equations presented so far) assumes ideal-solution behavior; thus, solute activity coefficients were omitted. This approximation is valid when low concentrations are used, especially when charged species are present. Thus, if the model were to be applied to concentrated systems, the explicit integration of activity coefficients would be required.

Osmotic water flux can be expressed in m s^–1^ as

12 $$J_{\mathrm{osm}}{=L}_{w}\times {\Delta \Pi =L}_{w}\times \Delta_{\mathrm{osm}}\times \mathrm{RT}\times 10^{3}$$

where L_w_ is the hydraulic conductivity coefficient in m s^–1^ Pa^–1^.

Most often, in the present context, L_w_RT is conveniently replaced by P_w_γ_w_ × 10^–3^, where P_w_ is the membrane permeability to water molecules in m s^–1^, molar volume γ_w_ is expressed in L mol^–1^, and multiplication by 10^–3^ is needed to convert to m^3^ mol^–1^, leading to^19,24,26,33^

13 $$J_{\mathrm{osm}}{=P}_{w}\gamma_{w}\times \Delta_{\mathrm{osm}}$$

As the vesicle swells, the increase in surface area introduces elastic stress in the membrane, generating a counter-pressure that damps water influx, according to Laplace law:^58–61^

14 $${\Delta P}_{\mathrm{el}}=\frac{2\sigma }{r}$$

where r is the vesicle radius and σ the membrane tension. The latter is given by

15 $${\sigma =K}_{A}\left(\frac{A_{v}-A_{0}}{A_{0}}\right)$$

which is the product of the area compressibility modulus, K_A_, and the areal strain. Here, A_0_ is the starting vesicle area at equilibrium.

Thus, the net water flux, J_w_, will be the result of the osmotic flux opposed by the elastic counter-flux, J_el_:

16 $$\begin{array}{c} J_{w}=J_{\mathrm{osm}}-J_{\mathrm{el}}=L_{w}\left(\Delta {\Pi -\Delta P}_{\mathrm{el}}\right)=L_{w}\left(\Delta_{\mathrm{osm}}\times \mathrm{RT}\times 10^{3}-\frac{{2K}_{A}}{r}\left(\frac{A_{v}-A_{0}}{A_{0}}\right)\right)=\\ =L_{w}\times {\mathrm{RT}\times 10}^{3}\left(\Delta_{\mathrm{osm}}-\frac{{2K}_{A}}{{r\times \mathrm{RT}\times 10}^{3}}\left(\frac{A_{v}-A_{0}}{A_{0}}\right)\right)=P_{w}\gamma_{w}\left(\Delta_{\mathrm{osm}}-\frac{{2K}_{A}}{{r\times \mathrm{RT}\times 10}^{3}}\left(\frac{A_{v}-A_{0}}{A_{0}}\right)\right) \end{array}$$

Eventually, water flow rate can be calculated as

17 $${Q=J}_{w}A_{v}\times 10^{3}$$

which has units of L s^–1^. The volume change was iteratively obtained by multiplying Q by the time step. This change was then applied to both the external and internal volumes with opposite signs. Finally, the updated V_in_ was used to adjust the vesicle diameter, d, and related quantities.

In the case of vesicle shrinking, the resulting negative membrane tension still satisfies Laplace law, reflecting the decrease in the vesicle’s apparent surface area. When the internal volume of giant unilamellar vesicles (GUVs; diameters ≥ 1 μm) decreases, their apparent surface area is reduced, which has been described as “projected surface area”. The total surface area remains nearly constant due to the accumulation of the excess area stored in membrane undulations and invaginations.^62–64^ Conversely, LUVs (with diameters on the order of hundreds of nanometers), which have a significantly higher area-to-volume ratio, do not exhibit these types of localized deformations. Instead, volume loss is accompanied by a global deviation from a spherical shape into an oblate or prolate spheroidal shape at constant total surface area.^58^ The resulting reduction in the apparent surface area can be described via a “sphere-equivalent surface area”. Consequently, vesicle shrinking is governed by bending energy. K_A_ in eq 16 is replaced by k_B_/Δx^2^, where k_B_ is the membrane elastic bending modulus (in J). It is worth anticipating here that the present model, for either swelling or shrinking, should be used only for low concentration gradients (≤100 mOsm/L with the vesicles considered in this study), as will be discussed below. In fact, important deviations can occur at larger gradients, including lysis, partial loss of contents, and additional energetic contributions to volume and area changes,^60,61^ which are beyond the scope of the present model.

The parameters *D* (eq 5) and *P*_*w*_ (eq 16) were systematically varied. At the end of each simulation, the corresponding volume-vs-time curve was obtained. To convert the simulated vesicle volume kinetics into the measured OD kinetics, we utilized a second-order polynomial approximation based on the RGD equation for light scattering. As demonstrated by Horner et al.,^26^ scattered light intensity against vesicle volume yields a smooth function lacking the inflection points present in radius-dependent plots, making its truncated Taylor series expansion highly accurate. The authors confirmed near-perfect overlap between the RGD model and the second-order polynomial approximation over a volume range matching the one pertaining to the present work, with volume variations up to 45%^26^ (our experimental volume changes remained constrained within ca. 16%; see below). The polynomial approximation introduces negligible mapping error while bypassing the computational and experimental burden of dynamically updating the form factor, the size parameters, and the internal refractive index. Thus, the relationship between vesicle volume and scattered light intensity (proportional to OD) can be conveniently represented by its truncated Taylor series expansion,^26^ and normalized OD data can be fitted to the normalized volume:

18 $$\frac{\mathrm{OD}\left(t\right)}{\mathrm{OD}\left(0\right)}=a+b\left(\frac{V_{\mathrm{in},\mathrm{eff}}\left(t\right)}{V_{\mathrm{in},\mathrm{eff}}\left(0\right)}\right)+c{\left(\frac{V_{\mathrm{in},\mathrm{eff}}\left(t\right)}{V_{\mathrm{in},\mathrm{eff}}\left(0\right)}\right)}^{2}$$

where V_in,eff_ is the effective internal volume obtained by taking into account even the molecular volume of the permeating species (although negligible under our conditions). Optimization of the fitting parameters was performed using the nonlinear least-squares Levenberg–Marquardt algorithm implemented in Gnuplot v6.0 (http://www.gnuplot.info/; accessed June 2026). To ensure physical consistency and mitigate parameter coupling, the water permeability, P_w_, and the zero-order polynomial coefficient, a, were fixed at 1 × 10^–6^ m s^–1^ and zero, respectively, according to the strategy discussed below. The remaining parameters, namely the polynomial coefficients b and c, and the solute diffusion coefficient D, were determined by minimizing the sum of squares of the residuals. To account for the experimental dead time and mixing, the fitting procedure was consistently initiated from t = 10 s, ensuring that the optimization focused on passive transport.

## Results and Discussion

A series of liposome swelling/shrinking assays (LSA) was performed using POPC LUV suspensions in the presence of gradients of KCl and standard amino acids to cover a broad permeability range. Thiourea was also included as a representative high-permeability solute. In all experiments, the maximum permeant concentration was limited to 20–25 mOsm/L. This modest value was chosen to maintain sufficient experimental sensitivity while ensuring that the method remains applicable to a wide panel of permeants, even those with limited solubility. Notably, the starting concentration gradients employed here are remarkably lower than those typically reported in the literature.^19,20,24,27,41,55^ This choice is critical from a modeling perspective, since, above specific membrane-tension limits, vesicles can undergo lysis or more complex burst-and-reseal mechanisms that result in the loss of internal content, a process difficult to model rigorously.^40,60–62^ In the case of vesicle shrinking, similarly, high gradients can introduce non-negligible energy contributions beyond simple membrane bending, leading to deviations from the ideal behavior described by basic mechanical models.^64^ Solution pH was monitored but not corrected, resulting in pH values of 6.0 ± 0.3 and 5.6 ± 0.3 before and after the measurements, respectively. Because the investigated amino acids lack ionizable side chains, the zwitterionic microspecies represents ≥ 99% of the total population across this pH interval (Table S1). The combined concentration of residual cationic and anionic species, and their impact on interfacial interactions, the osmotic gradient, and the membrane potential, are negligible.

The numerical analysis of the OD kinetics was performed following a multistep optimization protocol designed to ensure the uniqueness of the fit and mitigate parameter coupling. To define the optimal transport regime for the model, a sensitivity analysis was first conducted to evaluate the impact of water permeability, P_w_, on the relationship between the simulated vesicle volume and the experimental optical density. As shown in Figure S3, the mapping between V­(t)/V(0) and OD­(t)/OD(0) stabilizes for values of P_w_ ≥ 1 × 10^–6^ m s^–1^. Beyond this threshold, the optical response becomes effectively independent of the specific P_w_ value, as water flux is no longer the rate-limiting step for the observed kinetics. Consequently, P_w_ was fixed at 1 × 10^–6^ m s^–1^ for all subsequent simulations. From a physical standpoint, within the time scale of our experiments, solute translocation is the rate-limiting step. Fixing P_w_ at this threshold defines the lower boundary of the uncoupled transport regime. As such, the water permeability coefficient must be considered an apparent operational parameter for this specific experimental setting. However, it is important to note that the governing equations of the numerical model are physically grounded. Consequently, the model is capable of treating P_w_ as an independent, physically meaningful property whenever high-resolution kinetic data (e.g., from stopped-flow instrumental configurations) are supplied.

To further reduce the degrees of freedom in the curve fitting, the zero-order parameter of the polynomial approximation (eq 18) used to calculate OD­(t)/OD(0) from V­(t)/V(0) was fixed at zero. Finally, curve fitting was initiated at t = 10 s. This temporal offset allows the system to bypass the initial phase of solution mixing, thereby focusing data analysis on the pure passive diffusion regime, as shown by the series of blank kinetics under isotonic conditions in Figure S4.

Because our model simultaneously resolves the solute diffusion coefficient D through the membrane and the second- and first-order polynomial coefficients (eq 18) used to map volume changes to optical density, explicit identifiability testing is essential. Profile residual curves generated by scanning D over 6 orders of magnitude, while reoptimizing the polynomial coefficients, displayed a single global minimum (Figure S5) for all amino acids studied, confirming the uniqueness of the extracted parameters. A local profile scan (Figure S6) yielded a narrow 95% confidence interval in all cases, confirming that D and the corresponding permeability coefficients, P_S_, are locally identifiable and well constrained under the adopted experimental and modeling conditions.

### Nonpermeant Species

Potassium chloride was chosen as a virtually nonpermeant species. Reported permeability coefficients for passive, unassisted K^+^ and Cl^–^ transport through POPC-like lipid bilayers are ∼ 3.5 × 10^–14^ m s−1 ^27^ and ∼1.1 × 10^–12^ m s^–1^,^65^ respectively, at room temperature and neutral pH. Figure 1A shows the simulated volume kinetics resulting from curve fitting of the experimental results shown in Figure 1B, based on LSA performed with opposite starting gradients. All relevant parameters are reported in Table S2.

**Figure 1:**
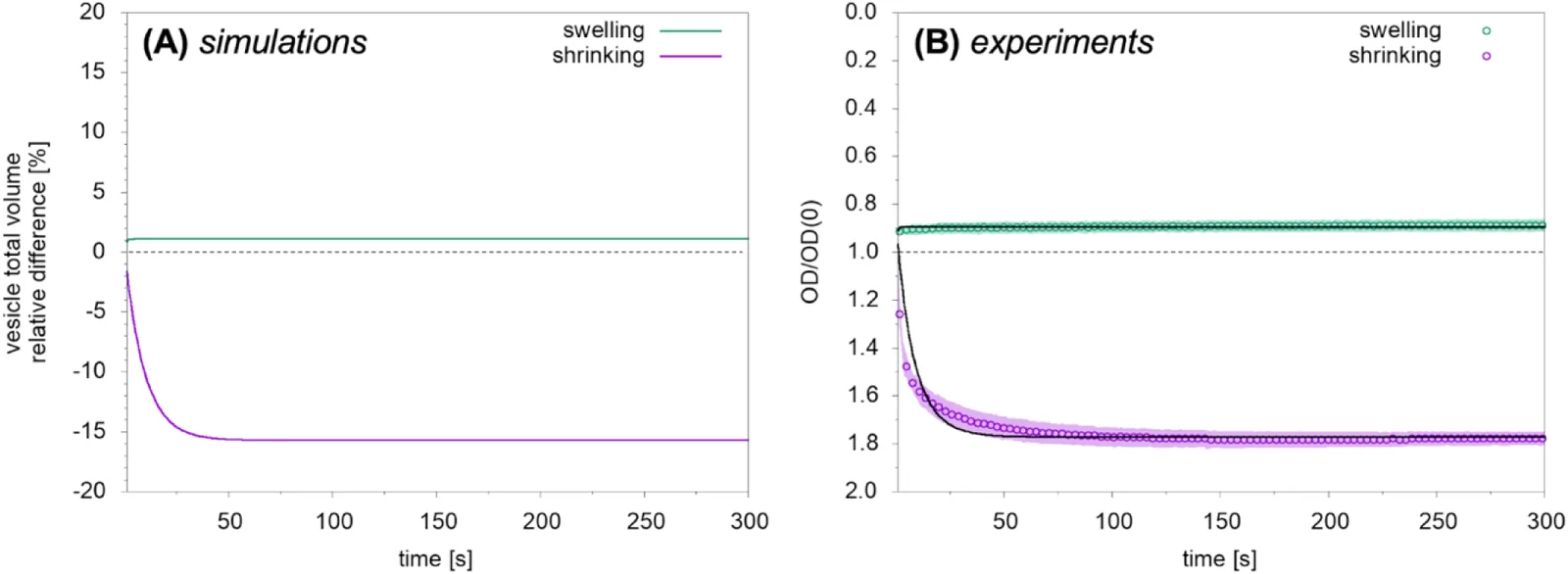
Simulated volume curves (A) resulting from the curve fitting of LSA results (B) for opposite KCl starting gradients (|Δ_osm_| = 19.77 mOsm/L). The curves highlight the asymmetric volume response of POPC vesicles to osmotic pressure (i.e., a greater resistance to swelling than shrinking) for a virtually nonpermeant species. Every third data point is shown for clarity. The shaded area indicates the point-by-point 2SD. OD(0) values were obtained from the corresponding isotonic experiments. Fitting curves are shown as solid black lines. For the sake of comparison with volume, the optical density scale has been inverted. All the relevant parameters are reported in Table S2.

In the case of nonpermeating species, water is the only substance that can move across the membrane. Depending on the sign of the initial osmolarity gradient, vesicles progressively shrink or swell, resulting in a proportional increase or decrease in optical density, respectively. In either case, the curve reached equilibrium within 300 s, as shown by the plateau.

Although the absolute value of the starting gradient was the same in the two experiments (Figure 1B), sensitivity is strikingly “asymmetric”. Experiments should be designed to exploit vesicle shrinkage. Such asymmetry in vesicle volume response, with much greater resistance to swelling than to shrinking, stems from general mechanical properties of lipid bilayers.^58–61,64^ As far as swelling is concerned, the area compressibility modulus of the lipid bilayer determines its resistance to changes in the equilibrium area per lipid. The reported values are large enough (200–235 mN/m for POPC at room temperature^63,66–68^ ) for the lipid bilayer to stretch only up to 3–5% before rupture (9–10 mN/m lysis tension^60^). This resistance is due to the high energy cost of progressive exposure of the hydrophobic core to water upon bilayer stretching. Conversely, in the case of shrinking, vesicles can accommodate volume loss at substantially lower energy cost. The reported elastic bending modulus is low enough ((10.5–12.1) × 10^–20^J for POPC at room temperature^66,69^ ) for the lipid bilayer to change shape without a significant variation in area per lipid. Volume loss is accommodated either by sequestering excess area, which accumulates in membrane undulations and invaginations in the case of GUVs,^64^ or through a constant-area deformation from a spherical to an oblate/prolate spheroidal shape in the case of LUVs.^58^ However, mechanical properties of lipid bilayers are seldom included in reported models for vesicle swelling/shrinking data analysis and permeability assessment.

Simulated curves in Figure 1A demonstrate the ability of the present model to reproduce the asymmetric vesicle response. Simulations reported a maximum areal strain as low as 0.7%, corresponding to a lipid bilayer tension of 1.6 mN/m. These values are significantly lower than the lysis limits reported in the literature.^60,61^ A systematic investigation showed that 115.6 mOsm/L is the concentration gradient at which a lysis tension of 9 mN/m is reached (Figure S7). Thus, we suggest that 100 mOsm/L is a precautionary limit. However, this may not be an on–off mechanism with a definite threshold, and the presented model was not designed to include this. Rather, a topologically continuous bilayer is assumed, which is inherently not applicable to vesicles undergoing swell-burst cycles. Thus, gradients as low as possible should be applied to reduce the probability of lytic events to the maximum extent possible. Conversely, significantly larger gradients were applied in previous studies, although possible burst-and-reseal mechanisms^58,59^ were not taken into consideration. Furthermore, when discussing lipid membrane permeability, the comparison of different methods, protocols, and even sample preparations is not trivial. In fact, membrane viscosity and elastic properties are largely affected by the specific solution composition, ultimately affecting permeability.^59,70–72^

Consistent with the preliminary sensitivity analysis (Figure S3), P_w_ was fixed at 1 × 10^–6^ m s^–1^ across all simulations. This value represents a transport regime in which water flux is no longer the rate-limiting step, allowing a clear separation of time scales between the initial mixing and osmotic stabilization, and the subsequent permeant solute-driven volume changes. While absolute determination of P_w_ for POPC LUVs requires rapid-mixing technologies to capture the fast kinetics reported in the literature (from 1.5 × 10^–4^ to 6.2 × 10^–5^ m s^–1^),^19,24,25,30^ the current fixed-threshold approach provides a reliable way to identify the membrane’s mechanical response and the permeability of the slower-diffusing species discussed in the following.

### Weak Permeant Species

Several standard hydrophobic amino acids were chosen. In the literature, permeability coefficients on the 10^–11^–10^–10^ m/s are reported for passive, unassisted transport at room temperature and neutral pH across the POPC bilayer.^55^ Vesicle shrinking experiments were analogous to those discussed above, conducted by replacing the external KCl with the amino acid while leaving the same small amount of KCl inside the vesicles. Water is not the only substance able to move across the membrane. Figure 2 shows the output from the simulation of phenylalanine taken as an example: three successive regimes can be clearly distinguished.

**Figure 2:**
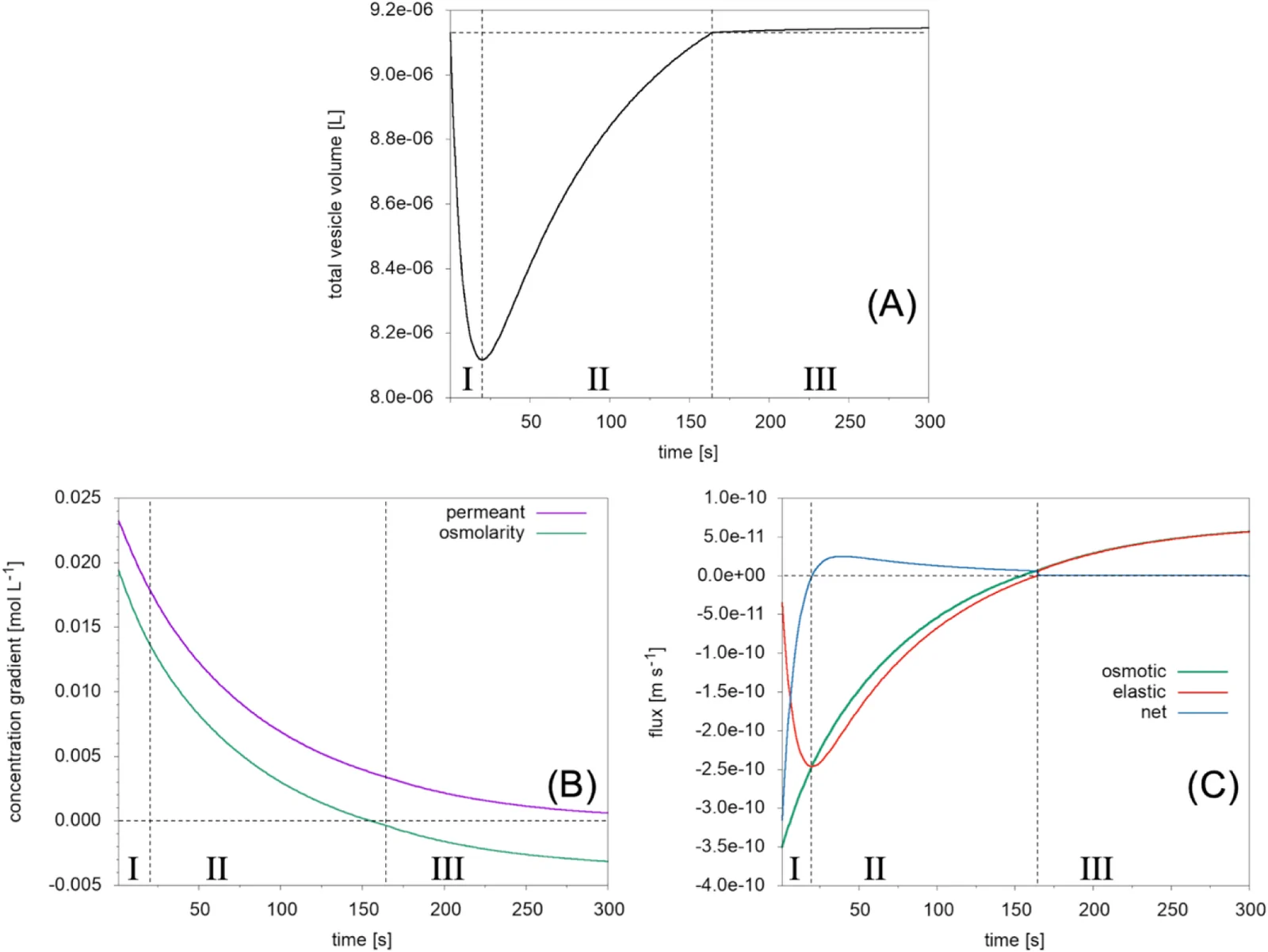
Output from the simulation for phenylalanine, representative of a weak permeant species: (A) total vesicle volume, (B) permeant and osmolarity gradients, (C) osmotic, elastic, and net flux densities are shown as a function of time. Parameters are listed in Table S3. Roman numerals are used to label the three consecutive stages of system evolution, separated by the dashed vertical lines: (I) an initial fast water efflux leading to significant vesicle contraction; (II) permeant influx causing internal osmolarity to increase and vesicles to slowly swell back to the starting volume; and (III) a shift to the swelling regime in which, due to the vesicles’ high resistance, volume variation levels off quickly.

At the beginning, both permeant and osmolarity gradients are at their maximum. They are comparable in magnitude and have the same sign, such that the net fluxes of the permeant and water molecules follow opposite directions: the permeant enters the vesicles, down its gradient; water molecules move out, toward the solution with the largest osmolarity. This first regime is dominated by the large membrane permeability to water, and the overall effect is a significant vesicle contraction. Due to the large difference between the external and internal volumes (V_out_/V_in_ ≅ 230), external permeant concentration remains virtually constant throughout the simulation, as already pointed out by other authors, who, in fact, simplified their models by neglecting its variation.^26,33,35^ Conversely, the internal osmolarity varies rapidly in the first regime, due to two contributions: (i) the incoming species, whose moles add to those of the internal nonpermeating osmolytes (NPO), and (ii) the leaving water, causing both the permeant and the NPO moles to become progressively concentrated inside the vesicles. However, elastic counterpressure progressively increases, up to the point when the net water flux becomes transiently zero, and volume variation reaches a minimum. The permeant’s concentration gradient is not fully dissipated at this point; it keeps flowing through the membrane into the vesicles during the second regime. Such mass transfer causes the internal osmolarity to further increase, allowing the system to progressively relax. Now, elastic pressure is slightly larger than the osmotic one, such that water slowly enters and swells the vesicles back toward the starting volume. During this stage, the system relaxation rate is determined by the permeant’s permeability coefficient. Eventually, the osmotic flux becomes transiently zero before permeant’s gradient is fully dissipated, due to the presence of the NPO inside the vesicles. The starting volume is reached, but the permeant still flows into the vesicles during the third regime. Its mass transfer is followed by that of water molecules, causing the system to switch from shrinking to swelling. However, due to the vesicles’ high resistance discussed above, the volume variation is quickly leveled off.

Figure 3 shows the simulated volume kinetics resulting from curve fitting of the experimental results from the LSA performed with a series of amino acids. All relevant parameters are reported in Table S3, and additional goodness-of-fit statistics are provided in Figure S8. With increasing permeant’s permeability coefficient, an OD maximum is clearly observed, and the slope of the curve after the maximum increases, resulting in a higher peak curvature. Moreover, both the peak and the final equilibrium are reached earlier.

**Figure 3:**
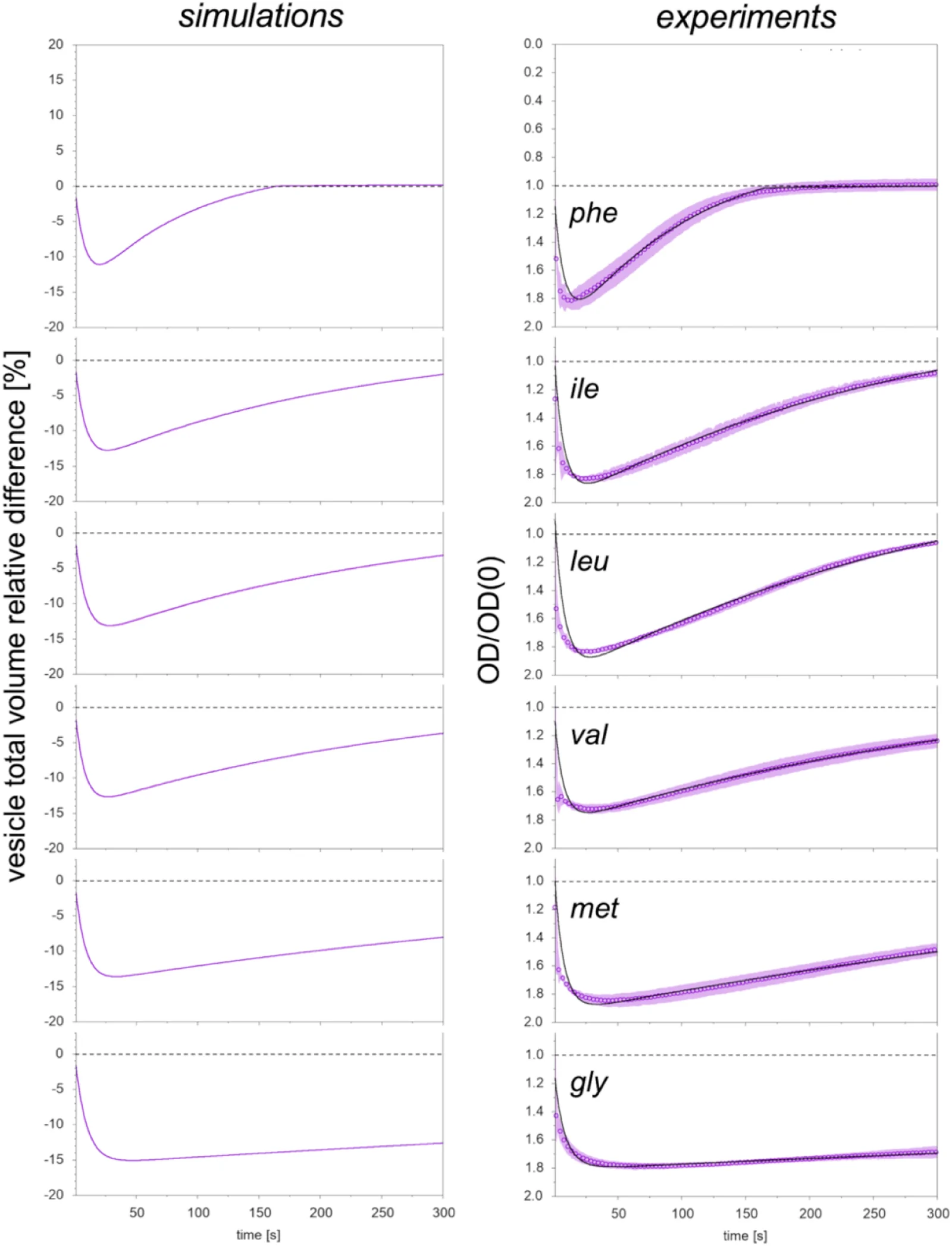
Simulated volume curves **(left)** resulting from the curve fitting of LSA results **(right)** for passive unassisted transport of several hydrophobic standard amino acids $\left({[S]}_{\mathrm{out}}-{[S]}_{\mathrm{in}}=23.\mathrm{26 mOsm}/L\right)$ . The data show that with an increasing permeant permeability coefficient, the OD maximum is observed earlier and the peak curvature increases. Every third data point is shown for clarity. The shaded area indicates the point-by-point 2SD. OD(0) values were obtained from the corresponding isotonic experiments. Fitting curves are shown as solid black lines. For the sake of comparison with volume, the optical density scale has been inverted. All the relevant parameters are reported in Table S3.

When permeant’s permeability coefficient is very low, water becomes virtually the only substance able to move across the membrane. Due to the initial osmolarity gradient, vesicles progressively shrink, resulting in an increase in optical density that eventually reaches the equilibrium plateau. In fact, with decreasing permeant’s permeability coefficient, the system’s evolution becomes progressively more similar to that obtained with nonpermeants (Figure 1).

Simulations demonstrate the ability of the present model to reproduce the experimental response to the weakly permeant species. Simulations indicate a maximum vesicle surface coverage as low as about 0.18%. Figure 4 and Table 1 provide an overview of the results.

**Figure 4:**
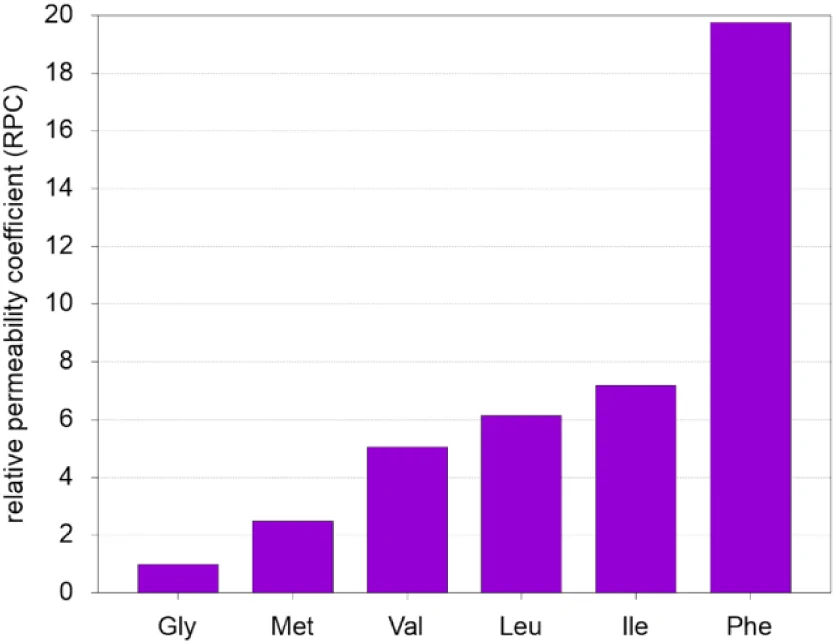
Relative (to glycine) permeability coefficients through the POPC bilayer are reported for the amino acids whose LSA curves are shown in Figure 3. The trend illustrates the expected increase in membrane permeability corresponding to the increasing hydrophobicity of the amino acid side chain.

**Table 1:** Main Results Obtained in This Work for the Six Selected Amino Acids^a^

**Amino acid**	^b^ **Log p**	^c^ **Diffusion coeff. [m** ^**2**^ **s** ^**–1**^]	^c^ **Permeability coeff. [m s** ^**–1**^]	^d^ **Permeability coeff. [m s** ^**–1**^]
Gly	–3.42	1.16 × 10^–13^	1.19 × 10^–11^	*not measured*
Met	–2.19	1.68 × 10^–14^	2.95 × 10^–11^	4.09 × 10^–11^
Val	–1.96	2.03 × 10^–14^	6.02 × 10^–11^	2.24 × 10^–11^
Leu	–1.59	1.06 × 10^–14^	7.32 × 10^–11^	4.54 × 10^–11^
Ile	–1.51	1.02 × 10^–14^	8.55 × 10^–11^	7.52 × 10^–11^
Phe	–1.19	1.34 × 10^–14^	2.35 × 10^–10^	6.48 × 10^–10^

a Permeability coefficients are compared with data available from the literature.

b Values from Chemaxon’s Chemicalize (https://chemicalize.com; Budapest, Hungary).

c This work.

d The literature value obtained through a stopped-flow, fluorescence-based methodology. Reproduced with permission from ref. 55. Copyright 2022 Springer Nature.

The correlation of these results with the physicochemical properties of the different permeants is discussed in the next section. Repeatability of both the diffusion and permeability coefficients is ± 6%, as evaluated by fitting the upper and lower bounds (mean ± 2SD) of the phenylalanine OD kinetics (Figure 3), which had the largest point-by-point SD. The close agreement between the permeability coefficients obtained with our label-free OD method and those reported by Frallicciardi et al.^35,55^ is highly encouraging, yet it is important to contextualize the fundamental differences between the two experimental approaches. State-of-the-art stopped-flow fluorescence protocols, such as that described by Frallicciardi et al., monitor volume changes indirectly by encapsulating self-quenching concentrations (around 10 mM) of organic fluorophores, such as calcein, inside the vesicles. Even neglecting possible deviations from the ideal solution approximation and interactions between these bulky, highly charged polyanionic molecules and either the phospholipids or the analyte, a time-consuming purification step via size-exclusion chromatography is mandatory to isolate the loaded vesicles from the unencapsulated dye, complicating the preparation protocol. In contrast, the presented approach reduces these complications by monitoring label-free, virtually unperturbed liposomes. Furthermore, while their advanced fluorescence analysis uses numerical differential equations to describe volume and area variations and considers vesicle size distribution, the presented model goes a step further by explicitly coupling mass transport to the lipid bilayer elasticity, distinguishing the mechanics of the swelling versus shrinking regimes.

It is worth noting that our current numerical model does not explicitly describe the individual contributions of a permeant’s different ionization states or possible specific interactions with the membrane surface. The latter are implicitly captured by the single macroscopic diffusion coefficient. To prevent model overparameterization, the experiments were deliberately performed at a pH where the single zwitterionic microspecies predominantly exists. While expanding the model to explicitly account for the full acid–base equilibria and multispecies transport would be a valuable future development, such an extension would require many discrete molecular descriptors that are often unavailable. Consequently, introducing these additional degrees of freedom would render the curve fitting underdetermined, unless specifically designed experiments were performed to quantify each parameter separately.

Finally, we present the simulation of a series of numerical experiments starting from isotonic conditions, with the permeant located inside the vesicles (Figure 5). All relevant parameters are the same as those listed in Table S3. In this way, one aims to exploit vesicle shrinking while eliminating the first stage of relatively fast water flow, making the experiment entirely rate-limited by the permeant’s permeability coefficient.

**Figure 5:**
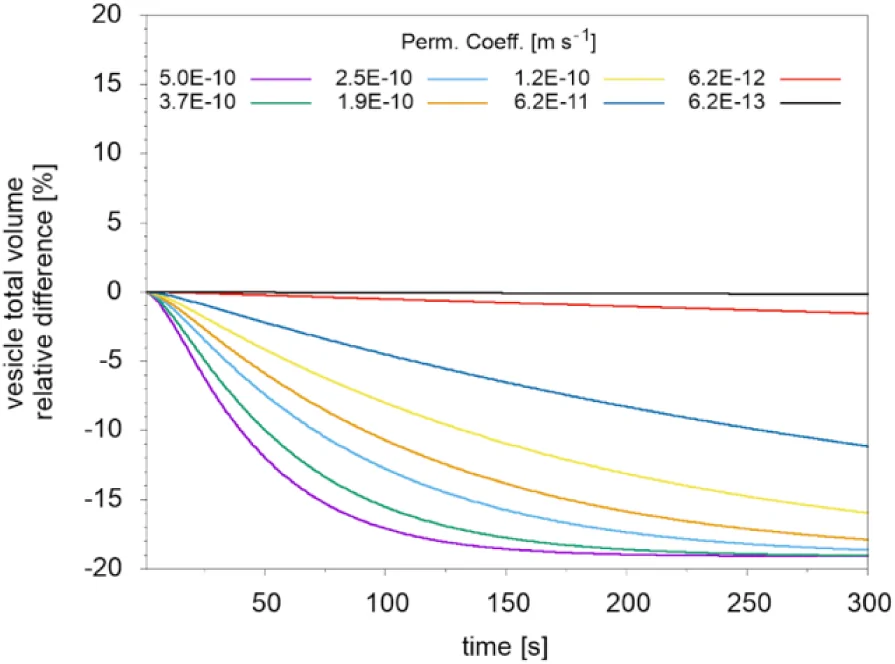
Examples of LSA volume kinetics as obtained from numerical simulations by setting the parameters reported in Table S3, starting from isotonic conditions, with the permeant species inside and nonpermeant osmolytes outside the vesicles $\left({[S]}_{\mathrm{in}}=25.\mathrm{00 mOsm}/L\right)$ . These simulations illustrate an alternative experimental setup aimed at exploiting vesicle shrinking while eliminating the initial fast water efflux stage.

This setup is not convenient without a proper rapid-mixing apparatus, because the largest difference in the slope of the curve is found at the beginning of the kinetics. Conversely, with the setup shown above, the permeant-dependent stage is shifted to longer times. Furthermore, both the position of the volume minimum and the time at which the plateau is reached (Figure 3) depend on the permeant’s permeability coefficient, providing additional, distinctive curve features that make curve fitting more robust. This is an example of how the numerical model can be used to rapidly check and compare different setups and conditions before moving to the laboratory bench.

It is also worth noting that, from a numerical point of view, different values of D and p can be chosen to obtain the same permeability coefficient (eq 6), although these would represent completely different physical scenarios. A given permeant might show poor membrane permeation either because of excessive hydrophilicity or low diffusivity through lipids. By describing the transport through the overall permeability coefficient alone, we cannot distinguish these two extremely different situations. One advantage of the current model is the explicit treatment of the partition equilibrium, which makes it possible to account for the relative weights of D and p in the transport process. However, it should be noted that the partition coefficients used herein represent calculated cheminformatic descriptors rather than experimentally measured membrane lipid partition coefficients. This choice aligns with standard practices in the pharmaceutical industry and, crucially, this approach eliminates the need for experimental partition measurements. Typically, the amount of permeant in the membrane phase is negligible compared with that in the aqueous phase. In this regime, the input partition coefficient and the extracted diffusion coefficient act as anticorrelated quantities. Numerical experiments confirmed that scaling the input partition coefficient up or down by 1 order of magnitude yields an exact reciprocal adjustment of the diffusion coefficient during curve fitting. This compensation leaves the absolute permeability coefficient completely invariant. Consequently, while individual D values should be considered apparent parameters correlated with the chosen lipophilicity scale, the absolute permeability and comparative rankings remain valid.

### Good Permeant Species

When the permeant’s permeability coefficient is larger than that of the amino acids shown in Figure 3, experimental sensitivity can decrease dramatically. The permeant species crosses the membrane quickly; thus, the volume peak becomes too small and occurs too early to be properly detected and analyzed with the current setup (Figure S9A). The shrinking regime (both the first and second stages, Figure 2) is short-lived, with most of the recorded kinetics occurring during the inherently insensitive swelling regime. Starting in isotonic conditions is not an option (unless rapid mixing is available). However, the experiment can be performed by starting with the permeant species inside the vesicles (Figure S9B). By starting with a highly permeant species in hypotonic conditions, only a small fraction of the kinetics occurs during the swelling regime, with the time needed to shift to the shrinking regime depending on the permeant’s permeability coefficient.

Thiourea was chosen as an example; a permeability coefficient of 5.52 × 10^–9^ m s^–1^ is reported for passive, unassisted transport through the membrane of human red blood cells at 295 K and neutral pH.^73^ Figure 6A shows the simulated volume kinetics resulting from curve fitting to the experimental results shown in Figure 6B, from both hypertonic and hypotonic LSA experiments, the former being analogous to those for the amino acids discussed above. All relevant parameters are reported in Table S4.

**Figure 6:**
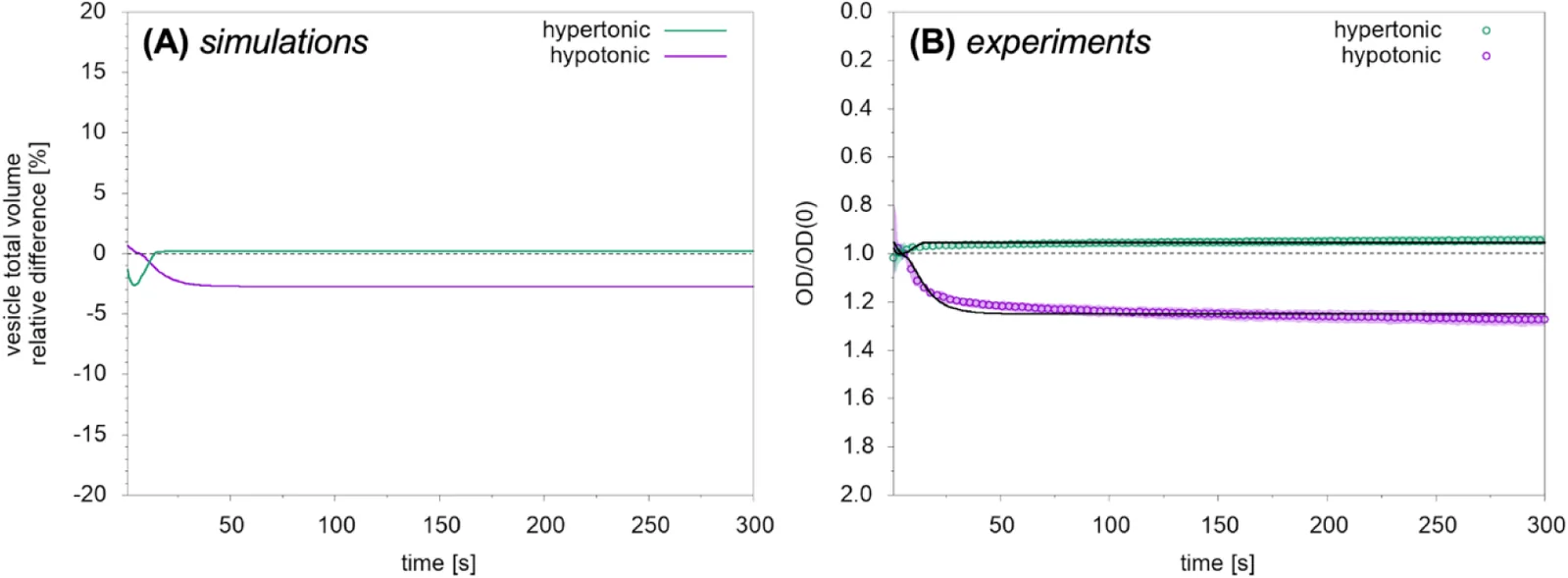
Simulated volume curves (A) resulting from the curve fitting of LSA results (B) for passive unassisted transport of thiourea, representing a good permeant species. Hypotonic conditions (with the permeant starting inside the vesicles) are shown alongside hypertonic conditions to demonstrate the improved experimental sensitivity achieved by shifting the majority of the kinetics into the shrinking regime. Every third data point is shown for clarity. The shaded area indicates the point-by-point 2SD. OD(0) value was obtained from the corresponding isotonic experiments. Fitting curves are shown as solid black lines. For the sake of comparison with volume, the optical density scale has been inverted. All the relevant parameters are reported in Table S4.

Simulations demonstrate the model’s ability to reproduce the experimental response and show how it can be used to test different experimental setups and conditions. The curve fitting of the experimental curve corresponding to the hypotonic condition gave a thiourea’s permeability coefficient of 5.62 × 10^–9^ m s^–1^ (RPC = 472), in remarkable agreement with the literature.^73^ Thiourea’s diffusion coefficient through the lipids was found to be 6.14 × 10^–14^ m^2^ s^–1^.

The expected strong positive correlation between permeability and hydrophobicity is confirmed (Figure 7A). Passive lipid membrane permeability for the investigated molecules is governed by the classic solubility-diffusion model, such that the higher the partition coefficient, the higher the molar fraction of membrane-bound permeant. This is the primary driving force for passive transport, generating an effective concentration gradient across the membrane.

**Figure 7:**
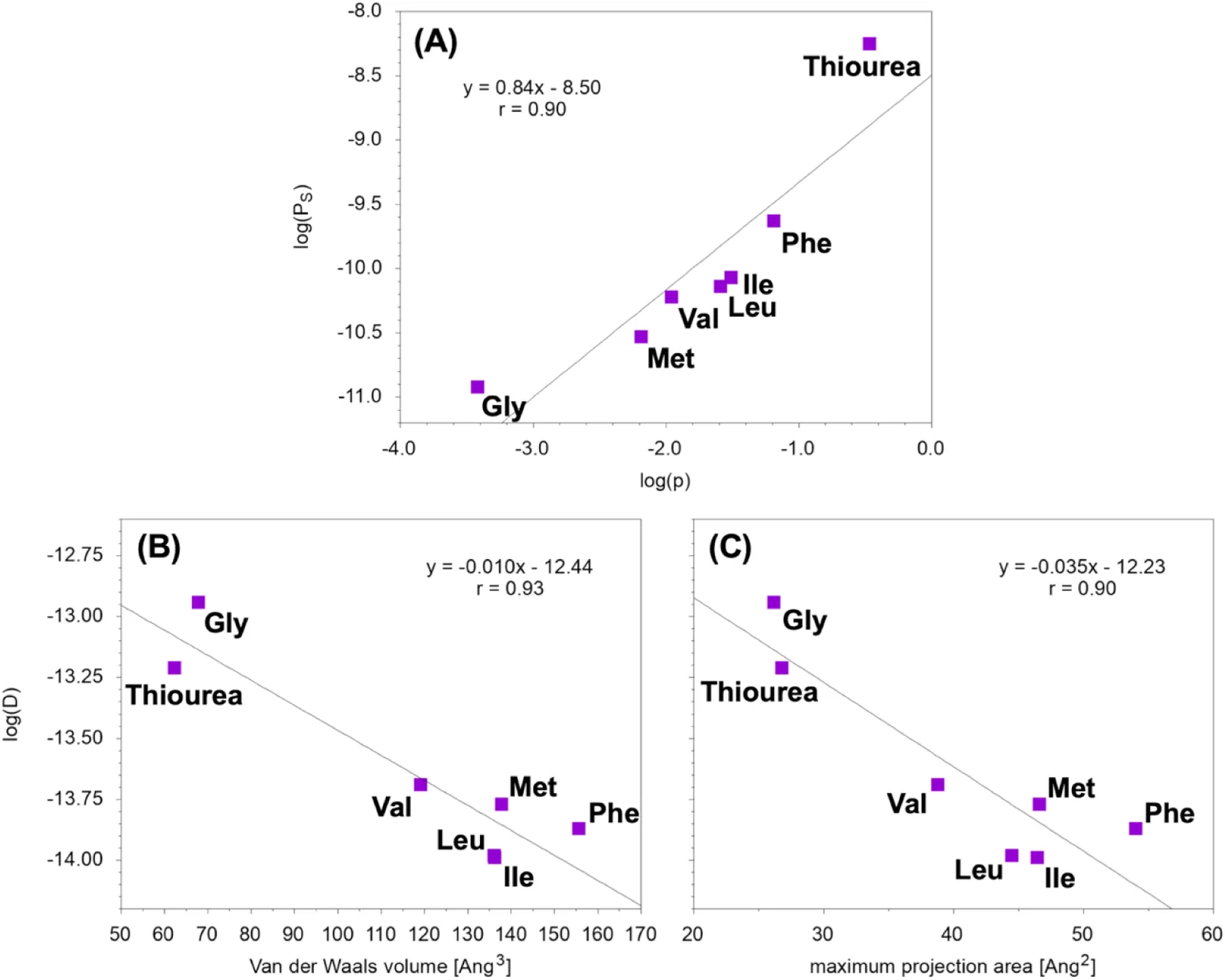
Correlations are shown for the results obtained in the present work by applying the model to curve fitting of experimental LSA kinetics (Figure 3 and 6), namely the logarithms of permeant’s (A) permeability and (B, C) diffusion coefficient, with molecular physicochemical descriptors obtained from Chemaxon’s Chemicalize (https://chemicalize.com; Budapest, Hungary), i.e.: (A) the logarithm of the partition coefficient, (B) van der Waals volume, and (C) maximum projection area. These correlations confirm the strong positive relationship between permeability and hydrophobicity and the strong anticorrelation between through-lipid diffusion and molecular size.

It is worth noting the very strong anticorrelation observed between the through-lipid diffusion coefficients of the investigated molecules and size descriptors (Figure 7B,C). This is an important result, showing that the presented algorithm discriminates between the weight of partition and diffusion to the permeation process.

## Conclusions

The developed computational model successfully simulates the dynamic behavior of unilamellar lipid vesicles under osmotic stress, enabling the determination of lipid bilayer permeability to various solute species over several orders of magnitude through the analysis of OD kinetics. The model is directly applicable to fluorescence data as well, by implementing literature-reported equations to describe relative fluorescence and its concentration dependence. A key feature of the presented model is the explicit treatment of the partition equilibrium and diffusion of the permeant through the membrane. The study reveals that the direction of net water transport can dramatically impact experimental sensitivity due to vesicles having greater resistance to swelling than to shrinking. This is extremely important and should be considered in the experimental setup, in addition to data analysis. The model can be used to test different setups and conditions in silico before moving to the laboratory bench. The algorithm can be directly extended to complex scenarios involving charge gradients, acid–base equilibria, multiple permeants, and even protein channels. The method is simple enough to be potentially applied within specifically designed high-throughput screening platforms that use microplate readers. Regarding prospective utility, while it is not designed to predict solute permeability directly from molecular topologies, it explicitly predicts the macroscopic volume kinetics and mechanical stress of vesicle suspensions under the specified initial transport conditions. Furthermore, by extracting permeability and diffusion coefficients from raw optical density data, this model can serve as a critical prerequisite for predictions, representing the source of the data sets to effectively train quantitative structure–property relationship (QSPR) and machine learning (ML) models for membrane permeation.

## Supplementary Material



## Data Availability

The data that support the findings of this study are available within the article and its Supporting Information and from the corresponding author upon reasonable request.

## Notes

Additional Figures S1–S9, showing numerical experiments aimed at verifying the impact of vesicle size distribution; sensitivity analyses to evaluate the impact of water permeability; blank isotonic control experiments; simulations of highly permeant species; and Tables S1–S4, with all the parameters for the simulations (PDF)
