# Supplementary material for: Modeling Lipid Vesicles’ Dynamic Response to Osmotic Pressure to Quantify Small-Molecule Transport
Source: Langmuir. 2026 Sep 29;42(40):29703–15. doi: 10.1021/acs.langmuir.6c02777 (PMC13674224; doi:10.1021/acs.langmuir.6c02777)
Supplement: Supplementary file 1 [file la6c02777_si_001.pdf]

Modeling lipid vesicles dynamic response to osmotic pressure to quantify small-molecule transport

Sara Boi,[a] Tiziana Podda,[b] Alessandra Murroni,[b] Stefan Milenkovic,[c] Matteo Ceccarelli,[c] Mariano Andrea Scorciapino[b,*]

[a]University of Cagliari, Department of Chemical and Geological Sciences, Monserrato (CA), I-09042, Italy.

[b]University of Cagliari, Department of Life and Environmental Sciences - Section of Drug Sciences, Monserrato (CA), I-09042, Italy.

[c]University of Cagliari, Department of Physics, Monserrato (CA), I-09042, Italy.

*corresponding author – marianoa.scorciapino@unica.it

SUPPLEMENTARY INFORMATION

Vesicle size distribution

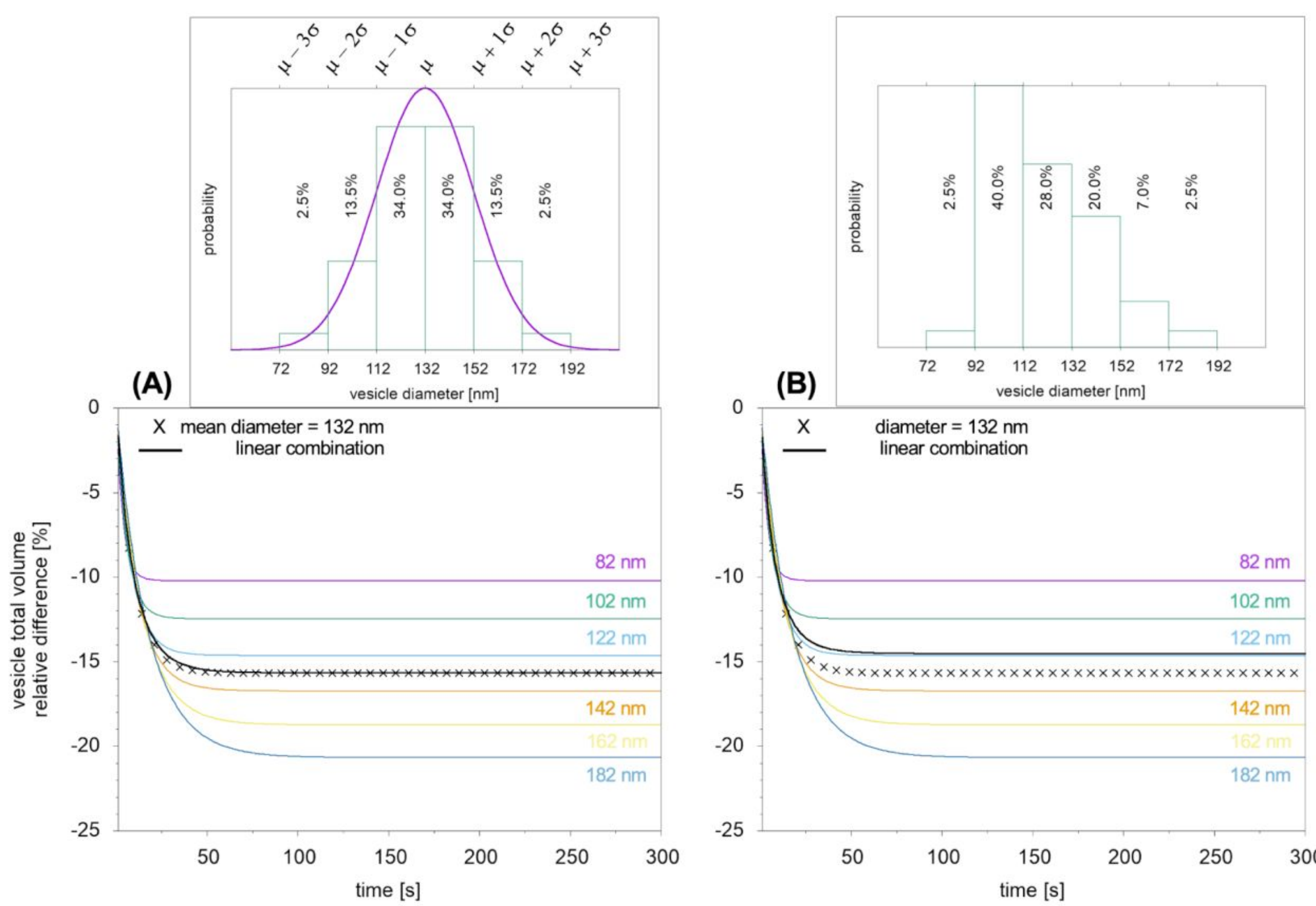

Figure S1. Simulated relative volume difference over time for a vesicle population with a Gaussian diameter distribution (**A**; μ = 132 nm, σ = 20 nm). Simulation parameters are those reported in Table S2 (shrinking). The distribution was discretized into bins for simplicity. Colored lines represent the simulations performed with individual vesicle diameters corresponding to each bin's midpoint. The solid black line represents the linear combination of these binned trajectories weighted by their respective probability, while the crosses (X) represent a single simulation performed using only the mean diameter (132 nm). In **(B)** a highly asymmetric discrete probability distribution is used to evaluate the deviation of total volume kinetics when the population symmetry is significantly altered.

The present model makes use of a single vesicle diameter value for simplicity, which we obtain as the average of the mean value of the size distribution from three DLS measurements. Figure S1 shows numerical experiments aimed at evaluating the impact of neglecting the size distribution. Figure S2 reports an example considering the typical experimental size distribution of our vesicle suspensions.

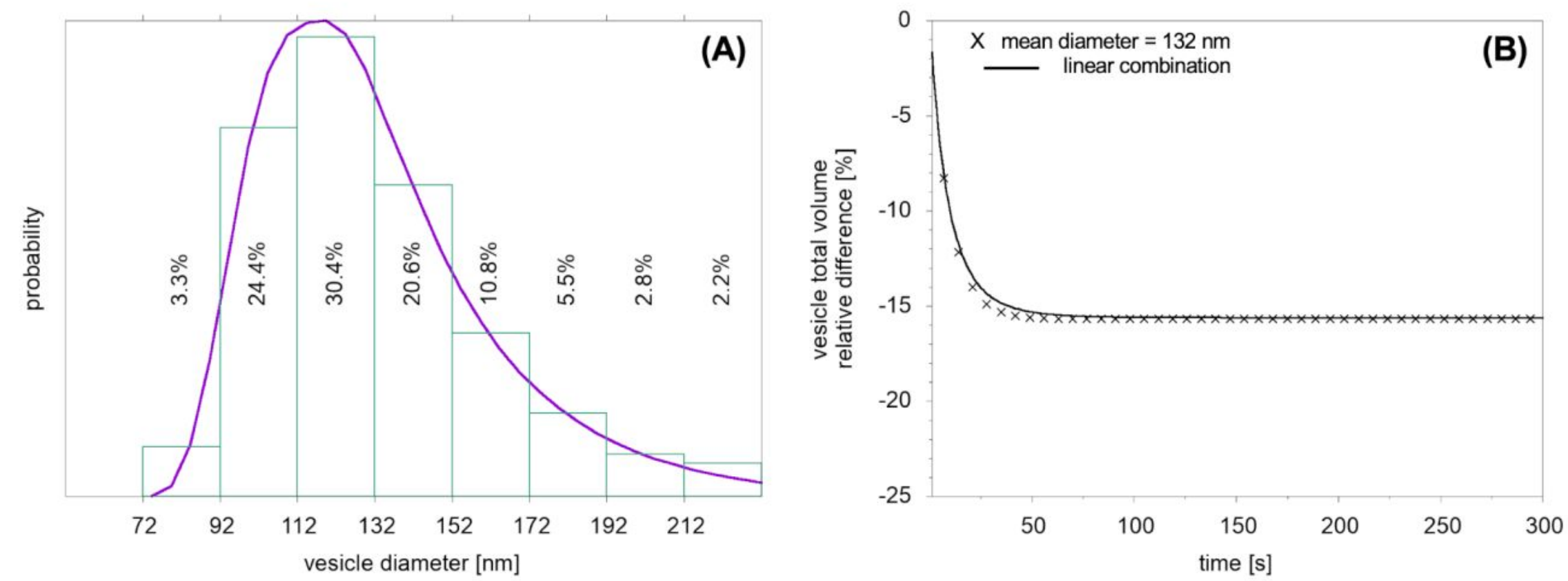

Figure S2. Example of our typical experimental vesicle size distribution (**A**; mean: 132 nm; mode: 119 nm; standard deviation: 30 nm). Simulation parameters are those reported in Table S2 (shrinking). The distribution was discretized into the same bins as in Figure S1 for comparison. (**B**) The solid black line represents the linear combination of the curves obtained from individual trajectories performed with individual vesicle diameters, corresponding to each bin's midpoint, weighted by their respective probability. The crosses (X) represent a single simulation performed using only the mean diameter.

These examples, demonstrate that vesicle size distribution can be neglected if it is not remarkably asymmetric, and that the mean value can be used to capture vesicle volume kinetics, in agreement with a previous report [12].

To provide a quantitative assessment of the error introduced into the curve-fitting by this approximation, a numerical analysis was executed. Independent simulations were run for each vesicle diameter bin as in Figure S2, by considering the representative case of leucine (mean diameter 126 nm; bin midpoints spanning 76 to 216 nm). Accordingly, a target diffusion coefficient, D, of $1.056 \cdot 10^{-14}$ m^2s^{-1} was applied. The resulting volume curves were converted into optical density by using the second-order polynomial approximation, and then linearly combined based on their respective experimental probabilities. This composite OD curve was then fitted by using the mean diameter approximation (126 nm). The fit converged to $D=9.935 \cdot 10^{-15}$ m^2s^{-1}. The resulting error is only -5.9%, confirming that the single-size model introduces a negligible bias.

Amino acids protonation equilibria

Table S1 reports the protonation equilibrium constants of the amino acids studied in this work, together with the pH measured before and after the liposome swelling/shrinking assay (LSA), and the molar fraction of the zwitterionic microspecies in this pH range, evaluated with the Henderson-Hasselbalch equation. The combined concentration of residual cationic and anionic species is negligible.

Table S1. Protonation equilibrium constants and the pH measured before and after LSA are reported for the amino acids studied in this work, together with the minimum molar fraction of the zwitterionic form in the same pH range.

**Amino acid**	**pK_a (carboxyl group)[a]**	**pK_b (amine group)[a]**	**pH before - after LSA**	**Molar fraction[b]**
Gly	2.35	9.78	6.13 - 5.64	0.998
Met	2.13	9.28	5.88 - 5.68	0.998
Val	2.29	9.74	5.26 - 5.39	0.998
Ile	2.32	9.76	6.14 - 5.89	1.000
Leu	2.33	9.74	6.00 - 5.71	0.998
Phe	2.20	9.31	6.14 - 5.94	0.999

[a]pK values from [13]; [b]minimum value obtained with the Henderson-Hasselbalch equation across the experimental pH range.

Sensitivity analysis of the relationship between volume and optical density

To justify the use of a universal polynomial mapping between the simulated relative volume, V(t)/V(0), and the experimentally measured relative optical density, OD(t)/OD(0), a systematic sensitivity analysis was conducted. This analysis aimed to determine the extent to which the assumed water permeability of the lipid bilayer, P_w, influences the shape of the optical response curve. By evaluating this relationship across a wide range of transport regimes it was possible to identify the limit where the numerical mapping becomes invariant, thereby ensuring the robustness of the subsequent curve fittings.

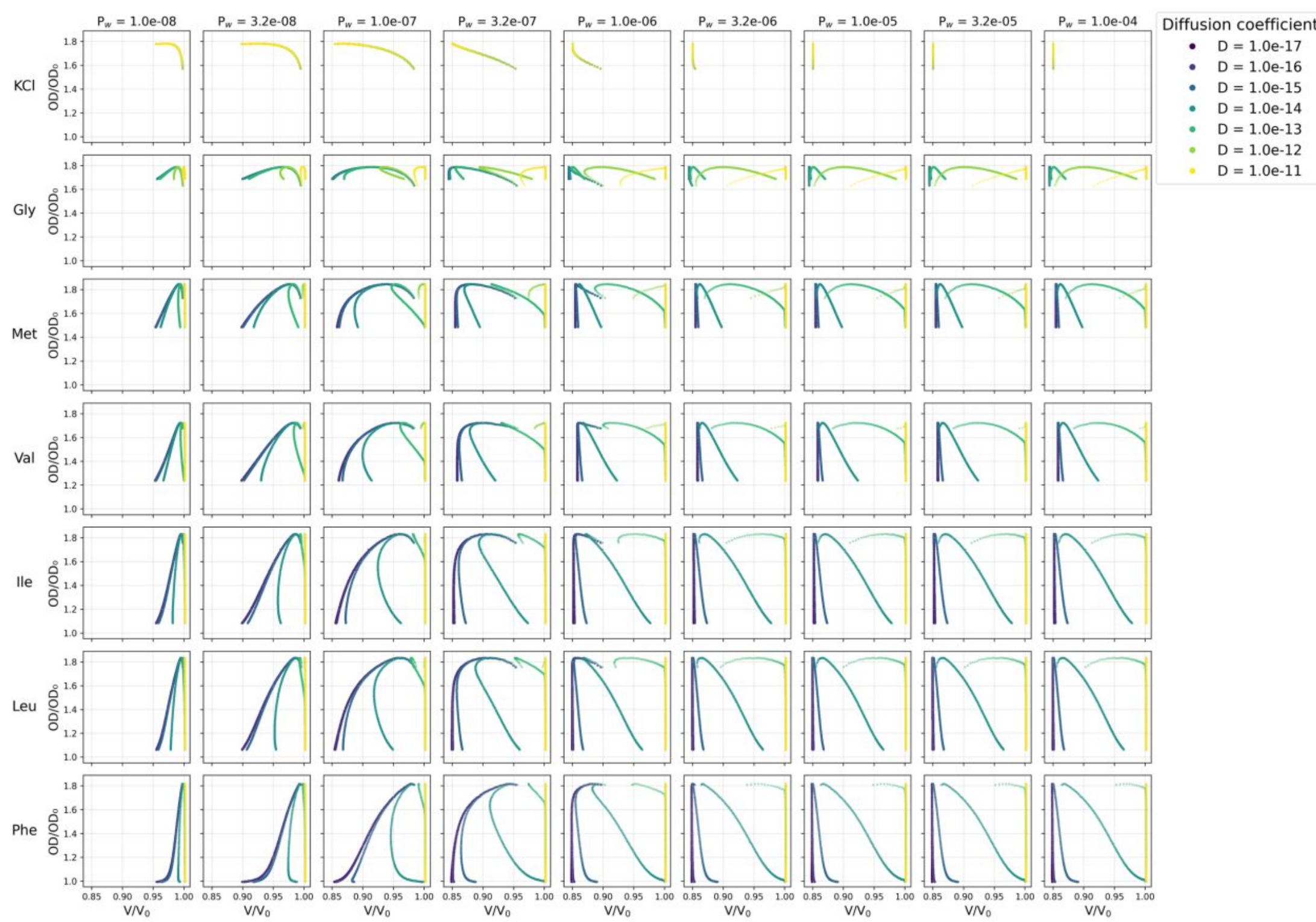

Figure S3. The relationship between the relative optical density, OD(t)/OD(0), and the simulated relative volume, V(t)/V(0), is shown for different solutes (KCl, glycine, methionine, valine, isoleucine, leucine, and phenylalanine). In each panel, the curves represent the mapping obtained by varying P_w from $1{\cdot}10^{-8}$ to $1{\cdot}10^{-4}$ m s^{-1}, and the solute diffusion coefficient, D, from $1{\cdot}10^{-17}$ to $1{\cdot}10^{-11}$ m^2 s^{-1}, as color-coded in the key. The analysis shows numerical convergence in the high-permeability regime ($P_w \geq 1{\cdot}10^{-6}$ m s^{-1}).

The sensitivity analysis reveals a clear convergence in the relationship between simulated volume and experimental optical density as a function of the assumed water permeability. At low P_w values, the mapping is significantly affected by the water flux rate. However, for

$P_w \geq 1\cdot10^{-6}$ m s^{-1}, the mapping for all the investigated species converges to a single, stable profile. This behavior indicates that once the water flux is sufficiently fast to be not the rate-limiting step of the global kinetic process, the V-to-OD mapping becomes independent of the specific P_w value, justifying the choice of fixing $P_w=1\cdot10^{-6}$ m s^{-1} in the following curve-fitting protocol.

This implies that above this value, the simulated OD curves for slow permeating species are insensitive to the exact water velocity. Therefore, $P_w=1\cdot10^{-6}$ m s^{-1} serves as a reliable numerical lower bound to simulate a water-insensitive kinetic regime, ensuring that the optimization routine can robustly extract the solute diffusion coefficient.

Blank experiments

Figure S4 shows 7 independent repetitions of a blank experiment (internal solution: KCl 3.75 mOsm/L; external solution: KCl 3.75 mOsm/L).

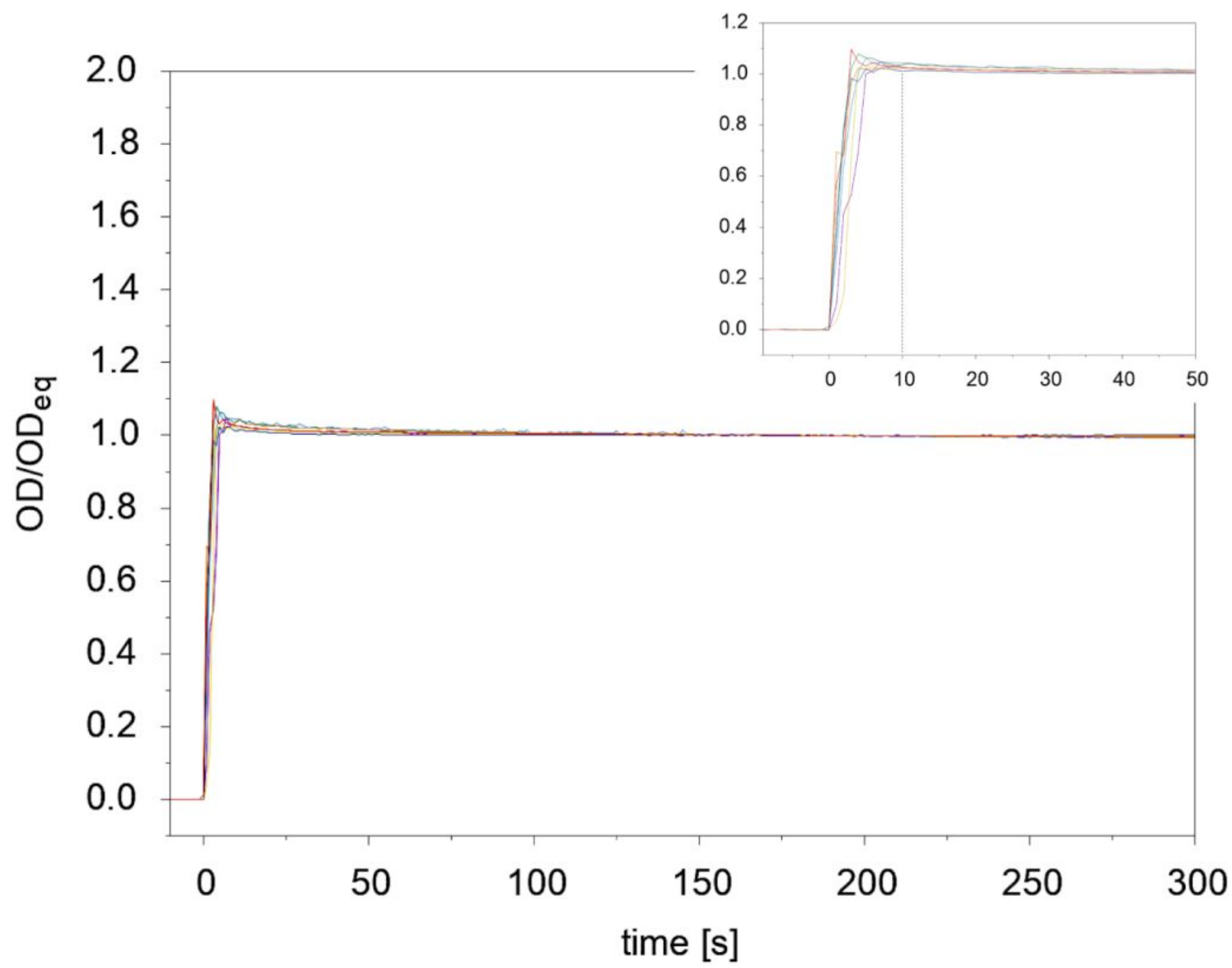

Figure S4. Optical density kinetics recorded for 7 independent repetitions of an isotonic experiment (internal solution: KCl 3.75 mOsm/L; external solution: KCl 3.75 mOsm/L). Curves were shifted to match the same LUV injection at 0 s. Data points have been scaled by the optical density value at equilibrium, OD_{eq}. The first part of the plot is zoomed in the inset.

In an isotonic experiment, OD rapidly increases after LUV injection, due to the vesicles scattering the incident beam. After a few seconds, the value level off, showing no further evolution and indicating complete solution mixing.

Parameter identifiability

To assess parameter identifiability and rule out non-uniqueness arising from coupling between solute transport and optical density mapping, a profile residual analysis was conducted. The fitting protocol remained identical to the main analysis: water permeability was fixed at $P_w=1\cdot10^{-6}$ m s^{-1}, the zero-order polynomial coefficient was fixed at a=0, and fitting was initiated at 10 s to exclude possible initial mixing artifacts. For each amino acid, the diffusion coefficient D was fixed at incremental values over a logarithmic grid spanning six orders of magnitude, while the optical density to volume mapping coefficients b and c were re-optimized via non-linear least squares. Profile residual curves (Figure S5) display a single, well-defined global minimum for all studied amino acids, confirming the uniqueness of the extracted parameters.

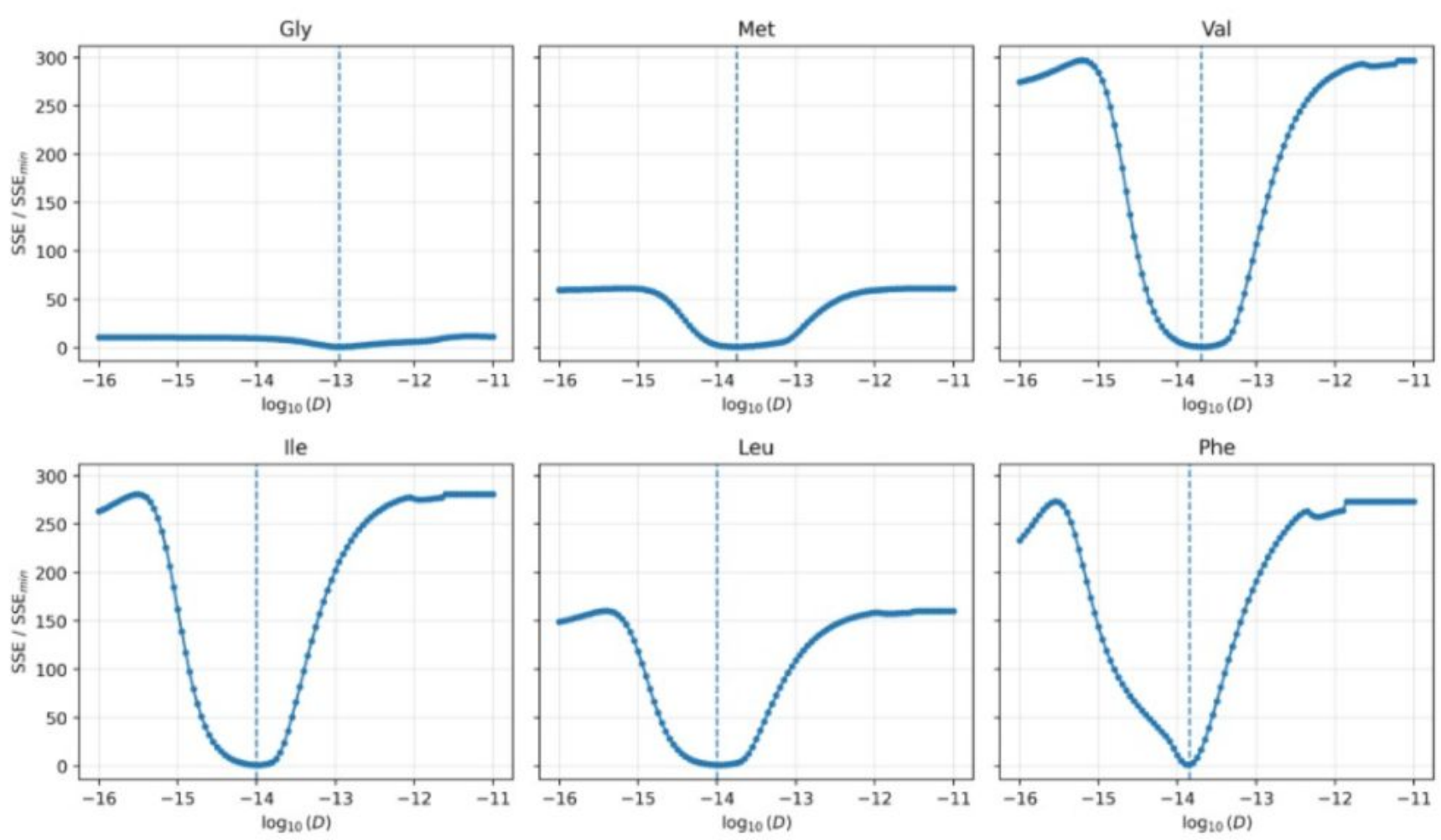

Figure S5. Profile residual analysis for solute diffusion-coefficient identifiability using a global logarithmic scan. For each amino acid (glycine, methionine, valine, isoleucine, leucine, and phenylalanine), the solute diffusion coefficient D was fixed at successive values over six orders of magnitude, while the optical density to volume mapping coefficients b and c were re-optimized under the defined fitting protocol ($P_w=1\cdot10^{-6}$ m s^{-1}; a = 0; fitting range t ≥ 10 s). The normalized residual sum of squares (SSE/SSE_{min}) is plotted against $\log_{10}D$. The vertical dashed line indicates the best-fit diffusion coefficient. In all cases, the residual profile exhibits a single, well-defined global minimum, confirming that the fitted diffusion coefficients are unique and locally identifiable.

Local dense grids were subsequently evaluated around each minimum to estimate profile-based 95% confidence intervals for D (Figure S6). A narrow 95% confidence interval was found in all cases, showing that D values and the corresponding permeability coefficients

P_S are locally identifiable and well constrained under the adopted experimental and modeling conditions.

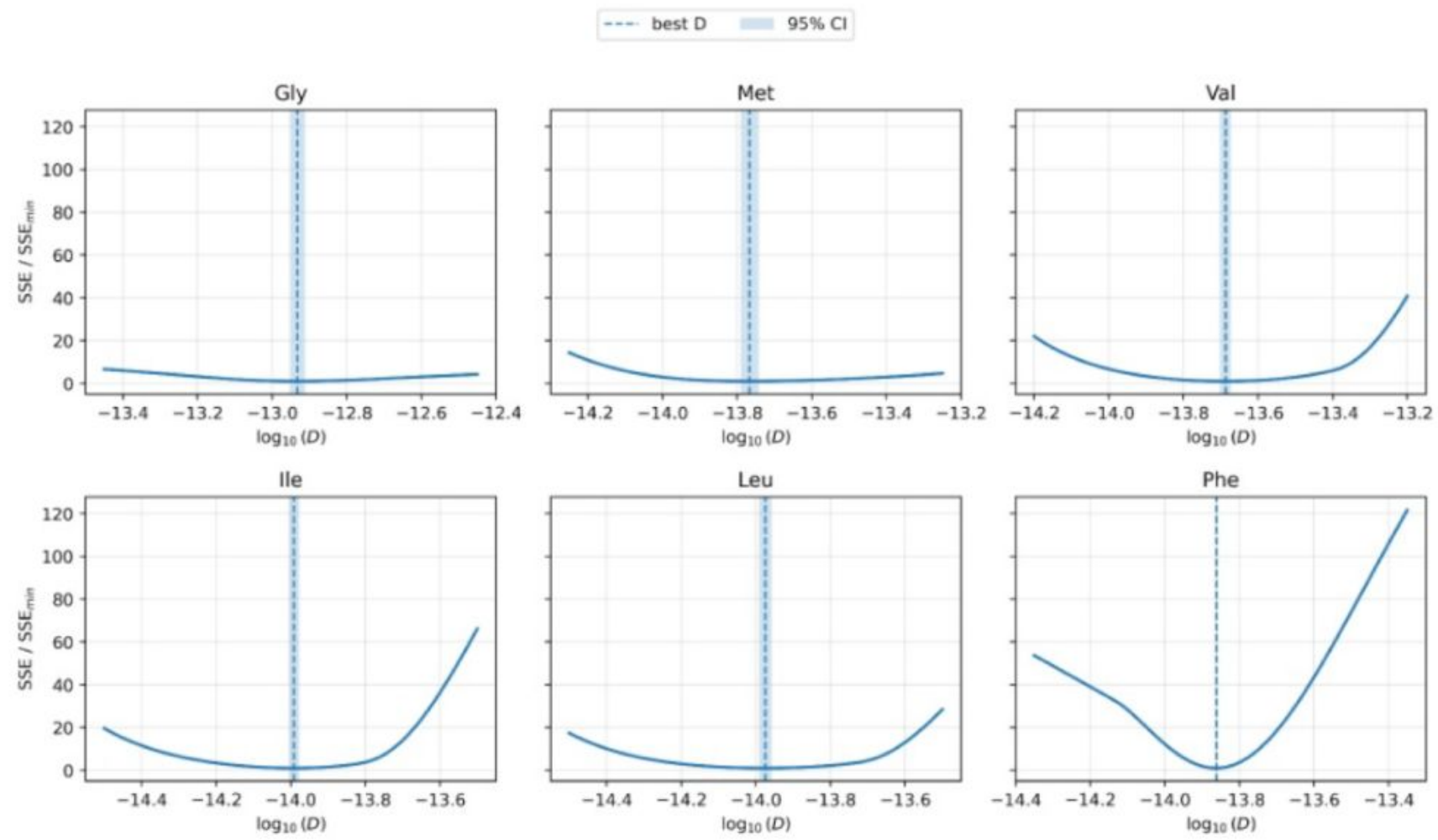

Figure S6. Refined profile residual analysis and approximate 95% confidence intervals for the fitted diffusion coefficient D. For each amino acid (glycine, methionine, valine, isoleucine, leucine, and phenylalanine), a dense local logarithmic scan of D was performed around the global optimum identified in Figure S5, re-optimizing the optical density to volume mapping coefficients b and c at each point. The normalized residual sum of squares (SSE/SSE_{min}) is plotted against $\log_{10}D$. The vertical dashed line marks the best-fit value of D, while the shaded region indicates the 95% profile-based confidence interval. The narrow 95% confidence interval demonstrate that the extracted D values are locally identifiable and well constrained under the adopted experimental and modeling conditions.

Non-permeant species

Table S2. Experimental and simulation parameters pertaining to the cases shown in Figure 1 of the main manuscript. When different values for a given parameter are associated to the shrinking or swelling case, these are explicitly labelled.

Parameter	Unit	Value
Temperature	K	$3.00 \cdot 10^{2}$
Lipid concentration	$mol\ L^{-1}$	$1.009 \cdot 10^{-3}$
Solution total volume	L	$2.150 \cdot 10^{-3}$
Average vesicles diameter	m	shrinking: $1.32 \cdot 10^{-7}$<br>swelling: $1.35 \cdot 10^{-7}$
[a]Lipid surface density [1–3]	m^{-2}	$1.38 \cdot 10^{18}$
Lipid molar volume [4]	$L\ mol^{-1}$	$7.62 \cdot 10^{-1}$
Lipid bilayer hydrophobic thickness [5,6]	m	$2.85 \cdot 10^{-9}$
[a]Bilayer area compressibility modulus [7–10]	$N\ m^{-1}$	$2.24 \cdot 10^{-1}$
[a]Bilayer elastic bending modulus [7,11]	J	$1.12 \cdot 10^{-19}$
Permeant's starting concentration in the external solution	$mol\ L^{-1}$	0
Permeant's starting concentration in the internal solution	$mol\ L^{-1}$	0
Additional starting osmolarity of the external solution due to non-permeant osmolytes	$Osm\ L^{-1}$	shrinking: $2.352 \cdot 10^{-2}$<br>swelling: $5.230 \cdot 10^{-3}$
Additional starting osmolarity of the internal solution due to a non-permeant osmolyte	$Osm\ L^{-1}$	shrinking: $3.750 \cdot 10^{-3}$<br>swelling: $2.500 \cdot 10^{-2}$
Water membrane permeability	$m\ s^{-1}$	$1.00 \cdot 10^{-6}$
Simulation time	s	$3.00 \cdot 10^{2}$
Simulation time-step	s	$1.0 \cdot 10^{-6}$
Output files writing frequency	$step$	$1.0 \cdot 10^{6}$

[a]Value has been obtained as the average of those reported in the quoted references.

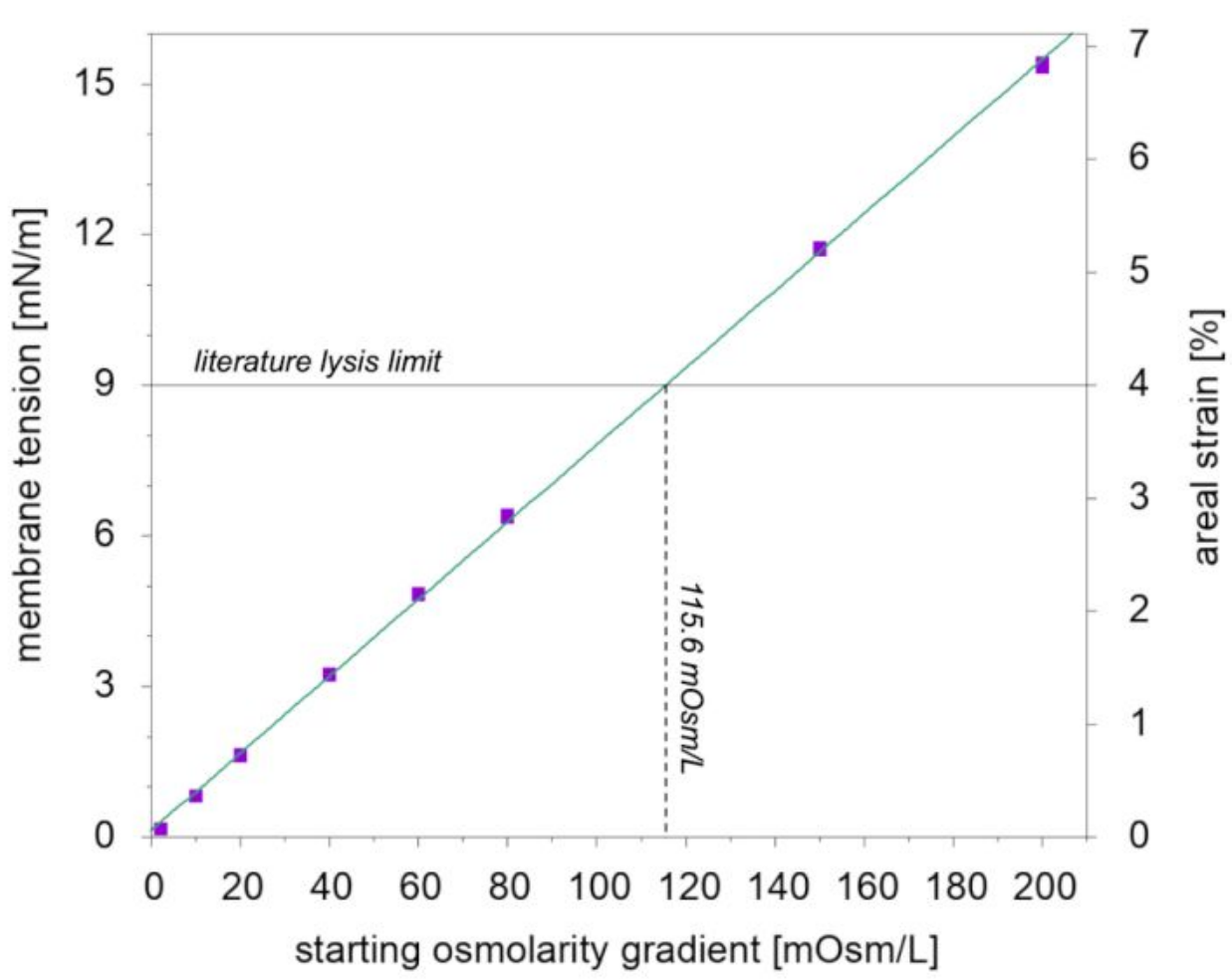

Figure S7. Membrane tension and the corresponding areal strain are shown for a series of simulations with non-permeant species at different starting gradients. The least-square linear fitting ($\sigma = 0.07677\ \Delta_{osm} + 0.1197$) shows a correlation coefficient of 0.99. The lowest limit for POPC vesicle lysis reported in the literature (see the main manuscript) is indicated by the horizontal straight line. Correspondingly, a theoretical upper limit of 115.6 mOsm/L for the starting gradient is identified.

Weak permeant species

Table S3. Experimental and simulation parameters pertaining to the cases shown in Figure 3 of the main manuscript. When case specific values are given to the same parameter, these are explicitly labelled.

Parameter	Unit	Value
Temperature	K	$3.00 \cdot 10^{2}$
Lipid concentration	$mol\ L^{-1}$	$1.009 \cdot 10^{-3}$
Solution total volume	L	$2.150 \cdot 10^{-3}$
Average vesicles diameter	m	phenylalanine: $1.27 \cdot 10^{-7}$<br>leucine: $1.26 \cdot 10^{-7}$<br>isoleucine: $1.24 \cdot 10^{-7}$<br>valine: $1.18 \cdot 10^{-7}$<br>methionine: $1.21 \cdot 10^{-7}$<br>glycine: $1.31 \cdot 10^{-7}$
[a]Lipid surface density [1–3]	m^{-2}	$1.38 \cdot 10^{18}$
Lipid molar volume [4]	$L\ mol^{-1}$	$7.62 \cdot 10^{-1}$
Lipid bilayer hydrophobic thickness [5,6]	m	$2.85 \cdot 10^{-9}$
[a]Bilayer area compressibility modulus [7–10]	$N\ m^{-1}$	$2.24 \cdot 10^{-1}$
[a]Bilayer elastic bending modulus [7,11]	J	$1.12 \cdot 10^{-19}$
Permeant's starting concentration in the external solution	$mol\ L^{-1}$	$2.326 \cdot 10^{-2}$
Permeant's starting concentration in the internal solution	$mol\ L^{-1}$	0
[b]Permeant's membrane partition coefficient		phenylalanine: $6.50 \cdot 10^{-2}$<br>leucine: $2.57 \cdot 10^{-2}$<br>isoleucine: $3.10 \cdot 10^{-2}$<br>valine: $1.10 \cdot 10^{-2}$<br>methionine: $6.50 \cdot 10^{-3}$<br>glycine: $3.80 \cdot 10^{-4}$
[b]Permeant molar volume (from the VdW volume)	$L\ mol^{-1}$	phenylalanine: $9.37 \cdot 10^{-2}$<br>leucine: $8.19 \cdot 10^{-2}$<br>isoleucine: $8.20 \cdot 10^{-2}$<br>valine: $7.17 \cdot 10^{-2}$<br>methionine: $8.30 \cdot 10^{-2}$<br>glycine: $4.09 \cdot 10^{-2}$
[b]Permeant maximum projection area	m^{2}	phenylalanine: $5.403 \cdot 10^{-19}$<br>leucine: $4.446 \cdot 10^{-19}$<br>isoleucine: $4.642 \cdot 10^{-19}$<br>valine: $3.876 \cdot 10^{-19}$<br>methionine: $4.661 \cdot 10^{-19}$<br>glycine: $2.615 \cdot 10^{-19}$
Additional starting osmolarity of the external solution due to non-permeant osmolytes	$Osm\ L^{-1}$	$2.616 \cdot 10^{-4}$
Additional starting osmolarity of the internal solution due to a non-permeant osmolyte	$Osm\ L^{-1}$	$3.750 \cdot 10^{-3}$
Water membrane permeability	$m\ s^{-1}$	$1.0 \cdot 10^{-6}$
Simulation time	s	$3.00 \cdot 10^{2}$
Simulation time-step	s	$1.0 \cdot 10^{-6}$
Output files writing frequency	$step$	$1.0 \cdot 10^{6}$

[a]Value has been obtained as the average of those reported in the quoted references.
[b]Value has been obtained from Chemaxon's Chemicalize (https://chemicalize.com; Budapest, Hungary, accessed in April 2026).

Goodness-of-fit statistics

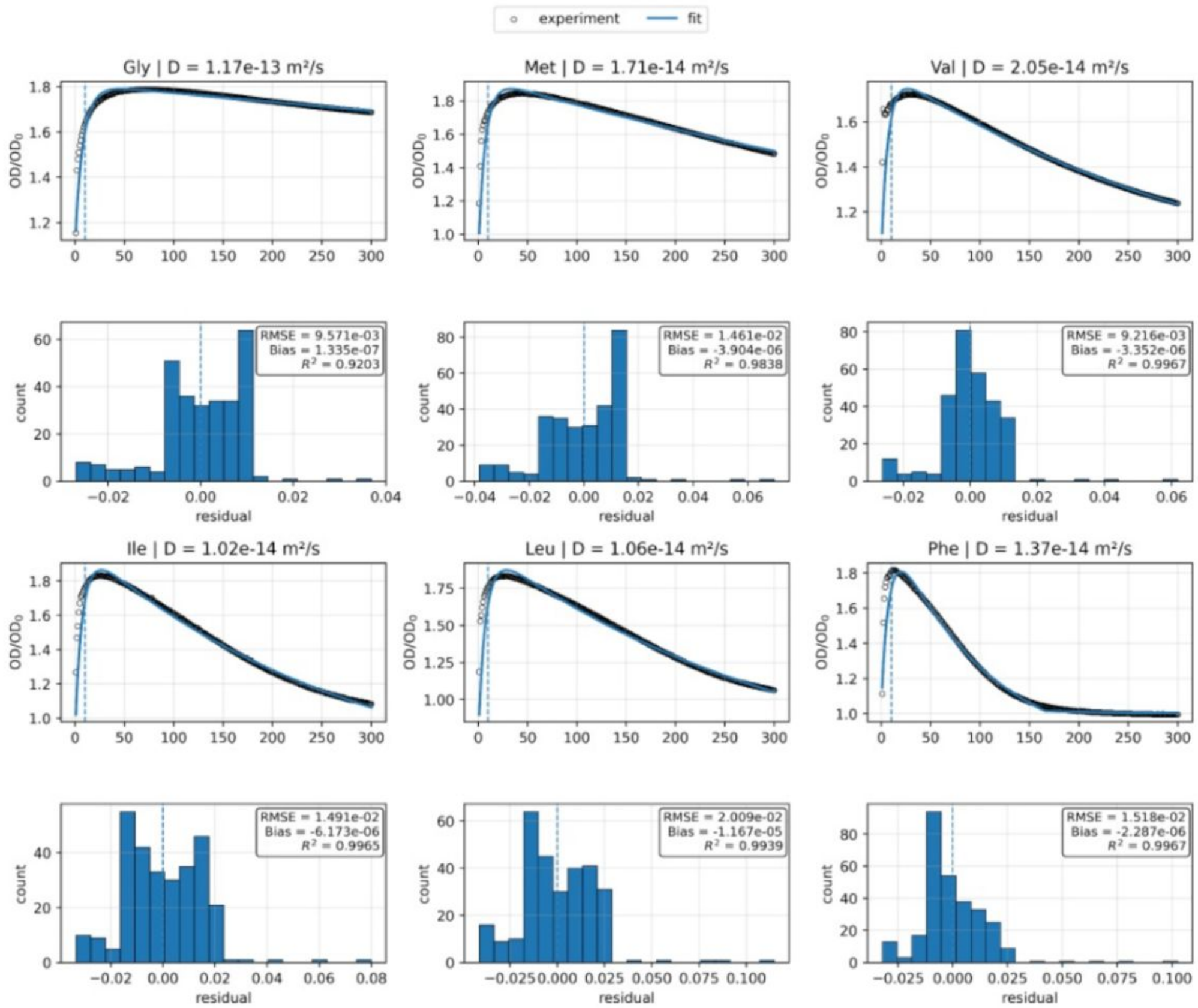

Figure S8. Best-fit optical density kinetics and residual distributions obtained for the six amino acids: glycine, methionine, valine, isoleucine, leucine, and phenylalanine. In each pair of panels: the experimental normalized optical density kinetics, OD(t)/OD(0) is shown **(top)**, together with the best-fit curve, and the vertical dashed line marks the fitting start at t = 10 s; the histogram of residuals for the data points included in the fit is reported **(bottom)**. The latter are centered close to zero and do not show marked or systematic asymmetry, supporting the adequacy of the fitting model. The corresponding RMSE, mean residual bias, and R^2 values are reported in each panel.

Good permeant species

Figure S9 shows simulated vesicle volume evolution in the case of a permeant species with membrane permeability coefficient larger than the amino acids discussed in the main manuscript. Simulations have been performed by considering the permeant species starting either outside or inside the vesicles. Simulation parameters are the same reported in Table S3.

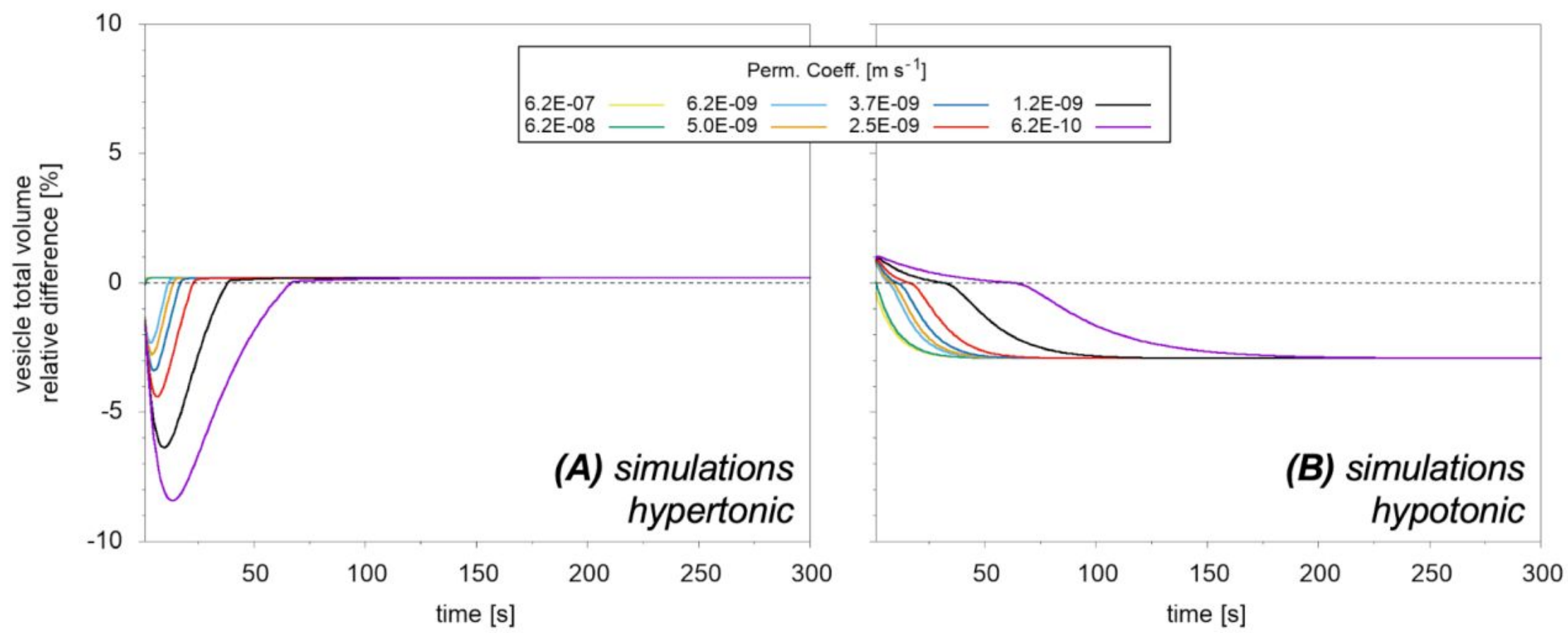

Figure S9. Examples of LSA volume kinetics as obtained from numerical simulations by setting the parameters reported in Table S3 and starting in either **(A)** hypertonic or **(B)** hypotonic conditions. Permeant's diffusion coefficient through the membrane was varied to obtain permeability coefficient values larger than those obtained for the investigate amino acids in Figure 3, namely: $1.0 \cdot 10^{-10}$; $1.0 \cdot 10^{-11}$; $1.0 \cdot 10^{-12}$; $8.0 \cdot 10^{-13}$; $6.0 \cdot 10^{-13}$; $4.0 \cdot 10^{-13}$; $2.0 \cdot 10^{-13}$; $1.0 \cdot 10^{-13}$ $m^2 s^{-1}$.

Table S4. Experimental and simulation parameters pertaining to the cases shown in Figure 6 of the main manuscript. When different values are given to the same parameter, these are explicitly labelled.

**Parameter**	**Unit**	**Value**
Temperature	K	$3.00 \cdot 10^{2}$
Lipid concentration	$mol\,L^{-1}$	$1.009 \cdot 10^{-3}$
Solution total volume	L	$2.150 \cdot 10^{-3}$
Average vesicles diameter	m	hypertonic: $1.31 \cdot 10^{-7}$<br>hypotonic: $1.16 \cdot 10^{-7}$
[a]Lipid surface density [1–3]	m^{-2}	$1.38 \cdot 10^{18}$
Lipid molar volume [4]	$L\,mol^{-1}$	$7.62 \cdot 10^{-1}$
Lipid bilayer hydrophobic thickness [5,6]	m	$2.85 \cdot 10^{-9}$
[a]Bilayer area compressibility modulus [7–10]	$N\,m^{-1}$	$2.24 \cdot 10^{-1}$
[a]Bilayer elastic bending modulus [7,11]	J	$1.12 \cdot 10^{-19}$
Permeant's starting concentration in the external solution	$mol\,L^{-1}$	hypertonic: $2.326 \cdot 10^{-2}$<br>hypotonic: $1.744 \cdot 10^{-3}$
Permeant's starting concentration in the internal solution	$mol\,L^{-1}$	hypertonic: 0<br>hypotonic: $2.500 \cdot 10^{-2}$
[b]Permeant's membrane partition coefficient		$3.39 \cdot 10^{-1}$

[b]Permeant molar volume (from the VdW volume)	$L\ mol^{-1}$	$3.75 \cdot 10^{-2}$
[b]Permeant maximum projection area	m^2	$2.676 \cdot 10^{-19}$
Additional starting osmolarity of the external solution due to non-permeant osmolytes	$Osm\ L^{-1}$	hypertonic: $2.616 \cdot 10^{-4}$<br>hypotonic: $3.488 \cdot 10^{-3}$
Additional starting osmolarity of the internal solution due to a non-permeant osmolyte	$Osm\ L^{-1}$	hypertonic: $3.750 \cdot 10^{-3}$<br>hypotonic: 0
Water membrane permeability	$m\ s^{-1}$	$1.0 \cdot 10^{-6}$
Simulation time	s	$3.00 \cdot 10^{2}$
Simulation time-step	s	$1.0 \cdot 10^{-6}$
Output files writing frequency	$step$	$1.0 \cdot 10^{6}$

[a]Value has been obtained as the average of those reported in the quoted references.
[b]Value has been obtained from Chemaxon's Chemicalize (https://chemicalize.com; Budapest, Hungary, accessed in April 2026).

References

1. Manzo, G.; Serra, I.; Pira, A.; Pintus, M.; Ceccarelli, M.; Casu, M.; Rinaldi, A.C.; Scorciapino, M.A. The Singular Behavior of a β-Type Semi-Synthetic Two Branched Polypeptide: Three-Dimensional Structure and Mode of Action. *Phys. Chem. Chem. Phys.* **2016**, *18*, 30998–31011, doi:10.1039/C6CP05464A.
2. Melo, M.N.; Ferre, R.; Feliu, L.; Bardají, E.; Planas, M.; Castanho, M.A.R.B. Prediction of Antibacterial Activity from Physicochemical Properties of Antimicrobial Peptides. *PLoS One* **2011**, *6*, e28549, doi:10.1371/journal.pone.0028549.
3. Maget-Dana, R. The Monolayer Technique: A Potent Tool for Studying the Interfacial Properties of Antimicrobial and Membrane-Lytic Peptides and Their Interactions with Lipid Membranes. *Biochim Biophys Acta* **1999**, *1462*, 109–140, doi:10.1016/s0005-2736(99)00203-5.
4. Santos, N.C.; Prieto, M.; Castanho, M.A. Interaction of the Major Epitope Region of HIV Protein Gp41 with Membrane Model Systems. A Fluorescence Spectroscopy Study. *Biochemistry* **1998**, *37*, 8674–8682, doi:10.1021/bi9803933.
5. Paula, S.; Volkov, A.G.; Van Hoek, A.N.; Haines, T.H.; Deamer, D.W. Permeation of Protons, Potassium Ions, and Small Polar Molecules through Phospholipid Bilayers as a Function of Membrane Thickness. *Biophys J* **1996**, *70*, 339–348.
6. Hills, R.D.; McGlinchey, N. Model Parameters for Simulation of Physiological Lipids. *J Comput Chem* **2016**, *37*, 1112–1118, doi:10.1002/jcc.24324.
7. Drabik, D.; Chodaczek, G.; Kraszewski, S.; Langner, M. Mechanical Properties Determination of DMPC, DPPC, DSPC, and HSPC Solid-Ordered Bilayers. *Langmuir* **2020**, *36*, 3826–3835, doi:10.1021/acs.langmuir.0c00475.
8. Weber, G.; Charitat, T.; Baptista, M.S.; Uchoa, A.F.; Pavani, C.; Junqueira, H.C.; Guo, Y.; Baulin, V.A.; Itri, R.; Marques, C.M.; et al. Lipid Oxidation Induces Structural Changes in Biomimetic Membranes. *Soft Matter* **2014**, *10*, 4241–4247, doi:10.1039/C3SM52740A.
9. Rawicz, W.; Olbrich, K.C.; McIntosh, T.; Needham, D.; Evans, E. Effect of Chain Length and Unsaturation on Elasticity of Lipid Bilayers. *Biophys J* **2000**, *79*, 328–339, doi:10.1016/S0006-3495(00)76295-3.
10. Doktorova, M.; LeVine, M.V.; Khelashvili, G.; Weinstein, H. A New Computational Method for Membrane Compressibility: Bilayer Mechanical Thickness Revisited. *Biophys J* **2019**, *116*, 487–502, doi:10.1016/j.bpj.2018.12.016.
11. Loftus, A.F.; Noreng, S.; Hsieh, V.L.; Parthasarathy, R. Robust Measurement of Membrane Bending Moduli Using Light Sheet Fluorescence Imaging of Vesicle Fluctuations. *Langmuir* **2013**, *29*, 14588–14594, doi:10.1021/la403837d.
12. Gabba, M.; Poolman, B. Physiochemical Modeling of Vesicle Dynamics upon Osmotic Upshift. *Biophysical Journal* **2020**, *118*, 435–447, doi:10.1016/j.bpj.2019.11.3383.
13. Voet, D.; Voet, J.G. Biochemistry, 4th ed. **2011**, John Wiley & Sons, Hoboken, NJ, USA.
